# Geometrically Tunable Scaffold‐Free Muscle Bioconstructs for Treating Volumetric Muscle Loss

**DOI:** 10.1002/adhm.202501887

**Published:** 2025-10-23

**Authors:** Bugra Ayan, Gaoxian Chen, Ishita Jain, Sha Chen, Gladys Chiang, Caroline Hu, Renato Reyes, Beu P. Oropeza, Ngan F. Huang

**Affiliations:** ^1^ Department of Cardiothoracic Surgery Stanford University Stanford CA 94305 USA; ^2^ Stanford Cardiovascular Institute Stanford University Stanford CA 94305 USA; ^3^ Veterans Affairs Palo Alto Health Care System Palo Alto CA 94304 USA; ^4^ Department of Chemical Engineering Stanford University Stanford CA 94305 USA

**Keywords:** geometrically tunable, modular, muscle regeneration, muscle tissue engineering, scaffold‐free, volumetric muscle loss

## Abstract

Traumatic muscle injuries associated with volumetric muscle loss (VML) are characterized by muscle loss beyond intrinsic regeneration capacity, leading to permanent functional impairment. Experimental therapies to augment muscle regeneration, such as cell injection, are limited by low cell transplantation capacity, whereas conventional engineered muscle tissue transplants lack geometric customization to conform to the shape of the muscle defect. Here, a facile approach to engineer scaffold‐free high‐density muscle tissues in customizable geometric shapes and sizes with high cell viability and integration potential is developed. Using a facile mold‐based approach to engineer scaffold‐free modular units, transcriptional profiling is performed to uncover the role of pre‐formed cell–cell interactions within scaffold‐free muscle bioconstructs on myogenesis, an the efficacy of muscle bioconstructs in a mouse model of VML is then evaluated. RNA sequencing revealed that pre‐formed cell–cell interactions supported myogenic pathways related to muscle contraction and myofibril assembly, unlike dissociated monodisperse cells. This work further demonstrates the therapeutic efficacy of 3D rectangular solid‐shaped scaffold‐free transplants in improving muscle function and vascular regeneration. Finally, toward clinical translation, the feasibility of this technology to integrate with medical imaging and artificial intelligence‐driven customized bioconstruct design and assembly for intraoperative use is illustrated.

## Graphical abstract

Volumetric muscle loss is associated with traumatic muscle resulting in permanent functional impairment. Mold‐based, scaffold‐free, high‐density muscle tissue bioconstructs are developed in customizable geometric shapes and sizes. The transplanted rectangular solid‐shaped muscle bioconstructs improved muscle force recovery and tissue regeneration in the ablated mouse muscle.

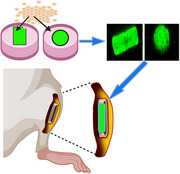

## 1 Introduction

Volumetric muscle loss (VML) is a critical condition characterized by the non‐recoverable loss of muscle tissue during traumatic injury,^[^ ^1^ ^]^ leading to persistent functional deficit.^[^ ^2^ ^]^ Conventional clinical interventions such as muscle flap transfer^[^ ^3^, ^4^ ^]^ or minced muscle tissue implants^[^ ^5^, ^6^ ^]^ prove suboptimal for treating extensive lower extremity injuries. Therefore, developing new strategies for muscle restoration and regeneration is an important unmet need.^[^ ^7^ ^]^ Tissue engineering emerges as a promising avenue for addressing damaged skeletal muscle underscored by inadequate endogenous regeneration,^[^ ^8^, ^9^ ^]^ while also enabling the study of tissue morphogenesis in vitro.^[^ ^10^, ^11^ ^]^ A number of tissue engineering approaches have been explored for treatment of VML in vivo. For example, moderate *de novo* formation of skeletal muscle was reported upon implantation of decellularized extracellular matrix (ECM) scaffolds.^[^ ^12^, ^13^, ^14^ ^]^ In addition, myogenic cell‐seeded ECM scaffolds have been shown to improve force generation and/or muscle regeneration at VML sites.^[^ ^15^, ^16^, ^17^ ^]^ Transplantation of ECM scaffolds containing myogenic cells in addition to support cells enhances muscle force production and/or tissue regeneration,^[^ ^18^, ^19^ ^]^ suggesting a therapeutic benefit of non‐muscle cells on muscle regeneration. Biophysical properties of the scaffold such as spatial patterning further have also been shown to modulate muscle regeneration and tissue organization.^[^ ^19^, ^20^, ^21^ ^]^ Although prior studies exploring scaffold‐free tissue engineering using molds to create geometrically defined bioconstructs remain limited,^[^ ^22^, ^23^, ^24^ ^]^ such studies demonstrate the potential of mold‐based strategies to generate modular tissues with controllable geometry and high cell density. However, full restoration of muscle function to pre‐injury levels remains elusive, as is their relevance for intraoperative use.^[^ ^13^ ^]^

Here we developed a modular scaffold‐free muscle tissue engineering strategy that supports high cell density, while enabling customizable geometries that can integrate with adjacent bioconstructs. The prefabricated modular muscle units were aspirated into VML defects to assess the regenerative potential of the engineered tissues for improvement in muscle function and vascular regeneration. Moreover, the scaffold‐free modular units were primed for myogenesis, as shown by transcriptomic profiling. These results highlight the promise of modular tissue engineering as a shape‐tunable and translationally relevant strategy for skeletal muscle regeneration.

## 2 Results

### 2.1 Biofabrication and Viability Analysis of Geometrically Customized Scaffold‐Free Modular Tissue Units

**Figure 1:**
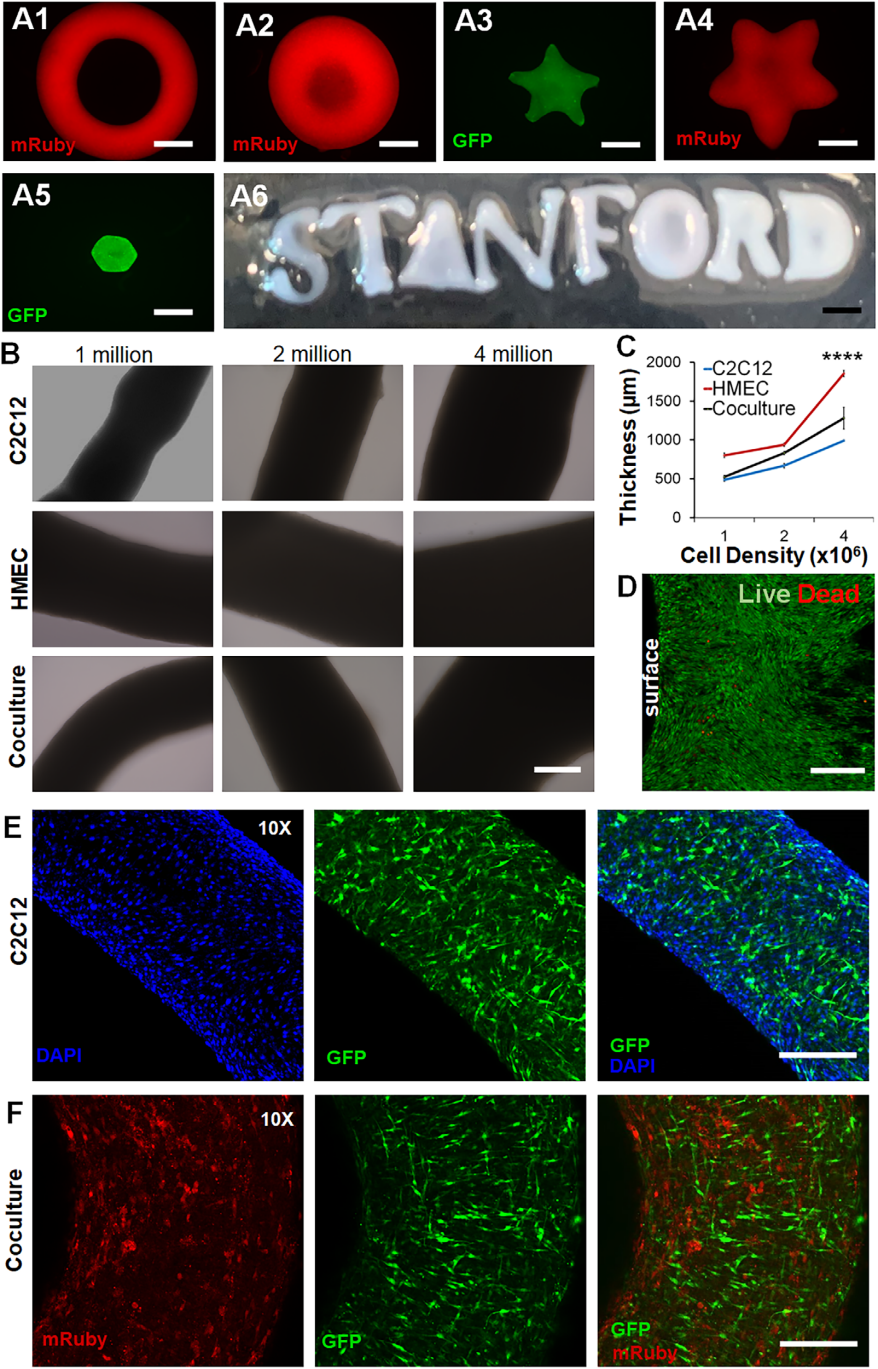
Biofabrication of scaffold‐free customizable modular shapes for tissue engineering. A) Biofabrication of scaffold‐free modular tissues comprising GFP‐labeled C2C12 myoblasts or mRuby‐labeled HMEC in the shapes of a toroid (A1), sphere (A2), stars (A3‐A4), hexagonal solid (A5), or alphabet letters spelling the word STANFORD (A6). B) Representative bright field images of modular tissue widths formed from either C2C12, HMEC, or co‐cultures in the form of rectangular solids. The initial cell seeding densities consisted of 1 million, 2 million, or 4 million cells. C) Quantification of modular tissue thickness after 2 days of biofabrication within molds (*n* = 3). D) Representative maximum intensity projections of confocal microscopy z‐stack images showing high cell viability, based on Live (green) and Dead (red) vital dyes. The image depicts the structure's surface at the left and increasing penetration depth along the right. E,F) Morphological comparison of rectangular solid modular tissues comprising C2C12 myoblasts alone (E) or in coculture with HMEC (F). Scale bars: A1‐A5 (200 µm); A6 (400 µm); B (400 µm); D (200 µm); E,F (400 µm). ****p<0.0001 indicates statistically significant comparison between 1 million and 4 million cells for each respective cell type (*n* = 3).

We developed a novel biofabrication method for the generation of scaffold‐free modular tissues for muscle tissue engineering. The tissue bioconstructs were generated entirely in molds and did not contain any exogenous biomaterials. The scaffold‐free tissue biofabrication method initiated with the 3D printing of customized shapes, followed by the formation of molds using agarose gels for cell seeding, and finally self‐assembly of the cells that conform to the shape of the molds (Figure S1, Supporting Information). To demonstrate the versatility of the scaffold‐free 3D biofabrication technology, we fabricated agarose molds comprising engineering tissue bioconstructs having diverse shapes and configurations in the mm size range. Using either green fluorescence protein (GFP)‐labeled C2C12 myoblasts or mRuby‐labeled human microvascular endothelial cells (HMEC) tissue constructs were successfully biofabricated in toroid, spherical, star, and hexagonal shapes using this approach (**Figure** **1**, A1–A5). Additionally, this method's capability was further highlighted by fabricating letter‐shaped tissue units and arranging them in a specific sequence to spell out the word “STANFORD” (Figure 1A6). The successful creation of complex, user‐defined shapes highlights the potential of the scaffold‐free biofabrication technique for generating intricate tissue architectures.

Scaffold‐free modular tissue units produce relatively high cell density tissue constructs, owing to the absence of exogenous scaffolds. To examine the impact of the initial cell seeding density on modular tissue construct dimensions, tissue constructs formed from rectangular solid molds were fabricated using C2C12 cells, HMECs, or a co‐culture of the two cell types at a 1:1 ratio. Where specified, the initial cell seeding concentrations ranging from 1 million to 4 million cells per tissue construct were evaluated (Figure 1B). The results showed that increasing the initial cell concentration resulted in a significant increase in the thickness of the biofabricated tissue construct after 2 days. For HMEC constructs, the thickness reached 1.86 ± 0.04 mm when fabricated with 4 million cells. In contrast, the thickness of C2C12 tissue constructs with 4 million cells was measured at 0.99 ± 0.01 mm (Figure 1C). The thickness of the co‐culture tissue structures with 4 million cells was at an intermediate thickness of 1.28 ± 0.14 mm. The observed difference in construct dimensions could be attributed to distinctive cell–cell interactions and the degree of contractility by each cell type, which consequently influenced the tissue compaction and organization during the biofabrication process.

To assess cell viability within the fabricated constructs, a viability assay was performed on the C2C12 tissue construct fabricated with 4 million cells (Figure 1D). Confocal imaging of z‐stacks through the thickness of the tissue construct revealed that the majority of cells within the construct remained viable, based on the robust uptake of the calcein‐AM (green) as an indicator of viable cells. The images showed scant numbers of non‐viable cells based on ethidium homodimer nuclear staining (red). This finding demonstrates the ability of the scaffold‐free biofabrication method to maintain cell viability even at high cell densities, an essential consideration for the generation of functional tissue constructs. Furthermore, to evaluate the cellular organization within the fabricated tissue constructs, confocal microscopy imaging was performed on the C2C12 and co‐culture tissue constructs derived from rectangular solid molds. The confocal images demonstrated that cells were uniformly distributed through the thickness of the tissue construct, and that in co‐culture, both populations showed uniform distribution (Figure 1E,F). In addition, we compared the viability of GFP‐labeled C2C12 myoblasts cultured within varying shapes for confocal analysis of cell viability, utilizing ethidium homodimer as a nuclear‐permeable dye for non‐viable cells (in red). Quantitative analysis of non‐viable cells through a depth of 300 µm demonstrates high cell viability of > 90% throughout the depth of analysis, regardless of cell shape (Figure S2, Supporting Information). The ability to fabricate tissue constructs with various shapes, sizes, and cell compositions, while maintaining cell viability and enabling the study of 3D cellular organization, by underscores the versatility of the scaffold‐free biofabrication method for high‐density culture of shape‐controllable tissue constructs.

### 2.2 Integration Potential of Modular Tissue Units In Vitro

**Figure 2:**
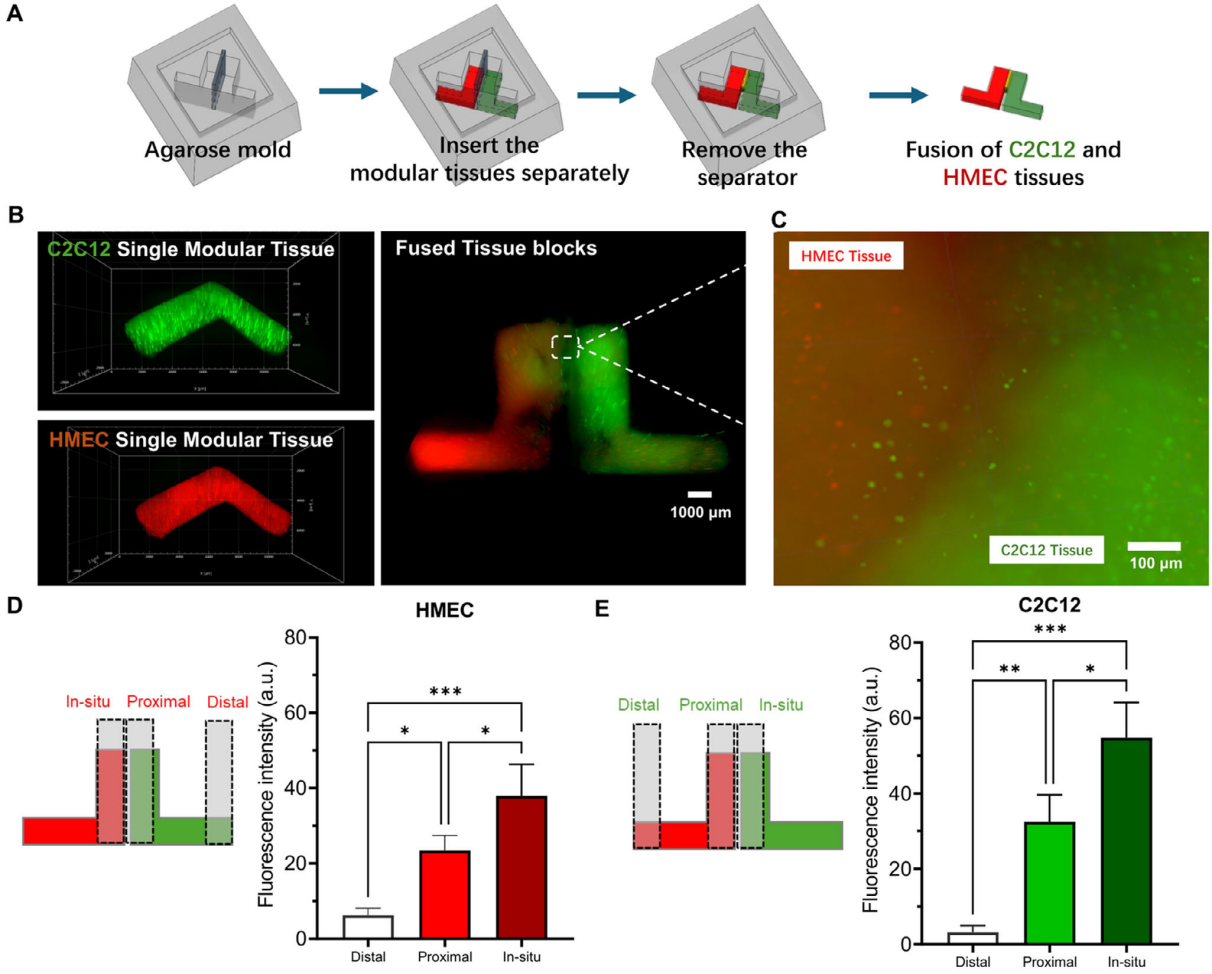
Integration of C2C12 and HMEC modular tissue units in vitro. A) Schematic illustration of the modular tissue fusion process. C2C12 (GFP‐labeled, green) and HMEC (mRuby‐labeled, red) modular tissues were embedded separately within an agarose mold, initially separated by a removable barrier. Upon removal of the barrier, direct contact facilitated cellular fusion over three days. B) Representative confocal fluorescence images showing independent C2C12 (green) and HMEC (red) modular tissues prior to fusion (left) and fused tissue blocks after 3 days of direct contact (right). Fluorescence overlap at the interface indicates cellular infiltration and integration. Scale bar: 100 µm. C) High‐magnification confocal imaging of the fusion interface reveals interpenetration of C2C12 and HMEC cells. Scale bar: 100 µm. D,E) Quantitative fluorescence intensity analysis of GFP (C2C12) and mRuby (HMEC) signals across three defined spatial regions: in situ (tissue region), proximal (adjacent to fusion boundary), and distal (peripheral region). Data are presented as mean ± SD (*p<0.05, **p<0.01, ***p<0.001, *n* = 3).

The feasibility for modular scaffold‐free units to integrate was next evaluated. The fusion between C2C12 (GFP‐labeled in green) and HMEC (mRuby‐labeled in red) tissue constructs was evaluated by confocal imaging, illustrating cellular interpenetration at the fusion interface. In the schematic sequence (**Figure** **2**A), C2C12 and HMEC L‐shaped modular units were positioned in close proximity within an agarose mold, separated initially by a removable barrier to ensure precise alignment. Following the removal of this barrier, a 3‐day incubation allowed direct cell contact, facilitating potential fusion. The 3D confocal reconstructions of independent L‐shaped modular units confirm that initially the GFP‐labeled C2C12 and mRuby‐labeled HMEC tissue units were distinct and separate (Figure 2B, Videos S1 and S2, Supporting Information). Upon fusion of the adjacent units for 3 days, the fused tissue unit (Figure 2B right, Video S3, Supporting Information) displayed notable GFP and mRuby fluorescence overlap at the interface, as highlighted at high magnification (Figure 2C). This finding suggests robust infiltration of fluorescently tagged cells across the fusion boundary. To quantify the degree of cellular infiltration of cells from one unit to the other, the integrated fluorescence intensity was measured in spatial regions of the fused tissue units (Figure 2D,E). For clarity of assessment, three spatial regions were defined: 1) the in situ region, representing the central location of the tissue unit; 2) the proximal region, representing the adjacent region to which cells migrate from one unit to the other tissue unit; and the distal region, located > 2 mm away from the interface where there is little intercellular migration to the other opposite tissue unit. Quantitative analysis of the integrated fluorescence intensity of each region revealed that, in fused tissues, the integrated GFP intensity from C2C12 cells in the proximal region representing the interface was 10.26 times higher than in the distal region (Figure 2E, *p*<0.01). Similarly, the corresponding integrated mRuby intensity from HMECs was 3.75 times higher in the proximal region than in the distal region (Figure 2D, *p*<0.05). These findings collectively confirm the successful establishment of cellular integration at the cellular level. Moreover, it suggests the potential for constructing multi‐unit tissue units for clinical‐scale applications, thereby supporting scalability and in vivo functional integrations.

### 2.3 Transcriptional Signature of Modular Muscle Units In Vitro

To demonstrate the efficacy of scaffold‐free modular tissue units to induce muscle regeneration in a mouse VML model, we first developed tissue units composed of C2C12 myoblasts, as they are a commonly used line of muscle myoblasts. However, the immortalized nature of these cells favor proliferation over differentiation, which may hinder efficient myogenesis in vivo. Therefore, we incorporated an in vitro pre‐differentiation period (either 2 days or 4 days) where the cells were initially cultured in conventional tissue culture flasks (2D culture) in differentiation media before 3D tissue biofabrication to augment myogenesis.

**Figure 3:**
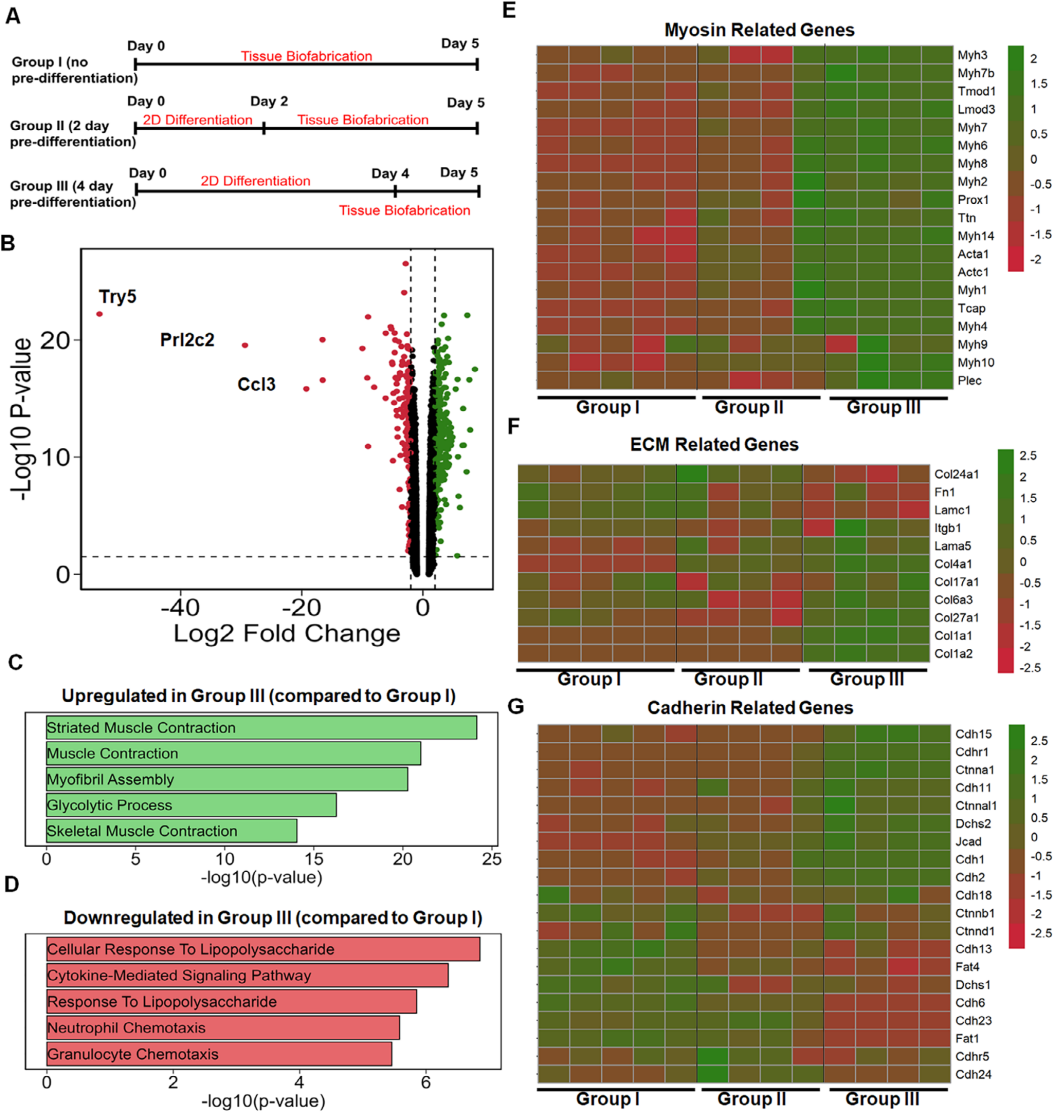
Transcriptional profiling of C2C12 modular tissue units with differential pre‐differentiation. A) Schematic of the experimental design of pre‐differentiation protocols in Group I, Group II and Group III. B) Volcano plot of fold‐change (x‐axis) and p‐value (y‐axis) of genes. Green: Upregulated (log2FC>2) in Group III versus Group I; Red: Downregulated (log2FC<‐2) in Group III versus Group I. C) Bar plot of gene sets enriched in upregulated genes in Group III versus Group I in the GO biological process database. D) Bar plot of gene sets enriched in downregulated genes in Group III versus Group I in the GO biological process database. E–G) Heatmaps of normalized counts for selective genes for Group I‐III (*n*≥ 3, P_adj_< 0.05).

To investigate the effects of pre‐differentiation prior to 3D tissue fabrication on muscle regeneration capacity, the tissue constructs formed from rectangular‐solid shaped molds were biofabricated using three distinct strategies, where the experimental groups were as follows: Group I: no pre‐differentiation; Group II: 2 days of pre‐differentiation; or Group III: 4 days of pre‐differentiation (**Figure** **3**A). To assess the effect of pre‐differentiation on myogenesis within the scaffold‐free 3D biofabricated C2C12 constructs, bulk RNA resequencing was performed (Figure 3). The effect of 2D pre‐differentiation before 3D tissue fabrication within the molds was notable, based on a significant downregulation of inflammatory markers (such as *Ccl3*, P_adj_ <10^−19^) and tumorigenic markers (such as *Prl2c2*, P_adj_<10^−23^) in the group that received pre‐differentiation (Figure 3B). Gene set enrichment analysis also revealed significant enrichment of muscle function and myosin assembly genes (P_adj_<10^−12^) that were upregulated in Group III, compared to Group I (Figure 3C). In particular, numerous myosin genes (including *Myh3*, *Myh1*, *Myh7b*) and myofiber assembly genes (such as *Tmod1*, *Tcap*, *Ttn*) were upregulated in Group III (Figure 3E), suggesting more advanced myogenesis in scaffold‐free biofabricated modular units. Conversely, downregulated genes in Group III were enriched in pathways related to Cytokine‐Mediated Signaling Pathways (P_adj_<10^−23^) and Cellular Response to Lipopolysaccharide (P_adj_<10^−4^), demonstrating a significant abrogation of inflammatory response with pre‐differentiation (Figure 3D). Additionally, ECMs such as collagens (*Col1a1*, *Col1a2*, and *Col4a1*), and laminin (*Lama5*) were also found to be upregulated in Group III compared to Group I as an indication of ECM remodeling associated with enhanced myogenesis in Group III (Figure 3F). Finally, there was also an upregulation of cell‐cell junction markers and ECM related genes including cadherins (*Cdhr1*, *Cdh1* and *Cdh2*) and catenins (*Ctnna1*, *Ctnnal1*) that were progressively upregulated from Group I to Group II to Group III, demonstrating an enhancement of 3D cell‐cell interactions with longer periods of pre‐differentiation. (Figure 3G). Together, these findings suggest that the incorporation of a 2D pre‐differentiation phase promoted improved tissue‐specific gene expression patterns and the expression of endogenous ECM components, ultimately contributing to the generation of more biomimetic engineered tissues.

### 2.4 Therapeutic Testing of Pre‐Treated C2C12 Modular Muscle Units in Mouse VML Model

**Figure 4:**
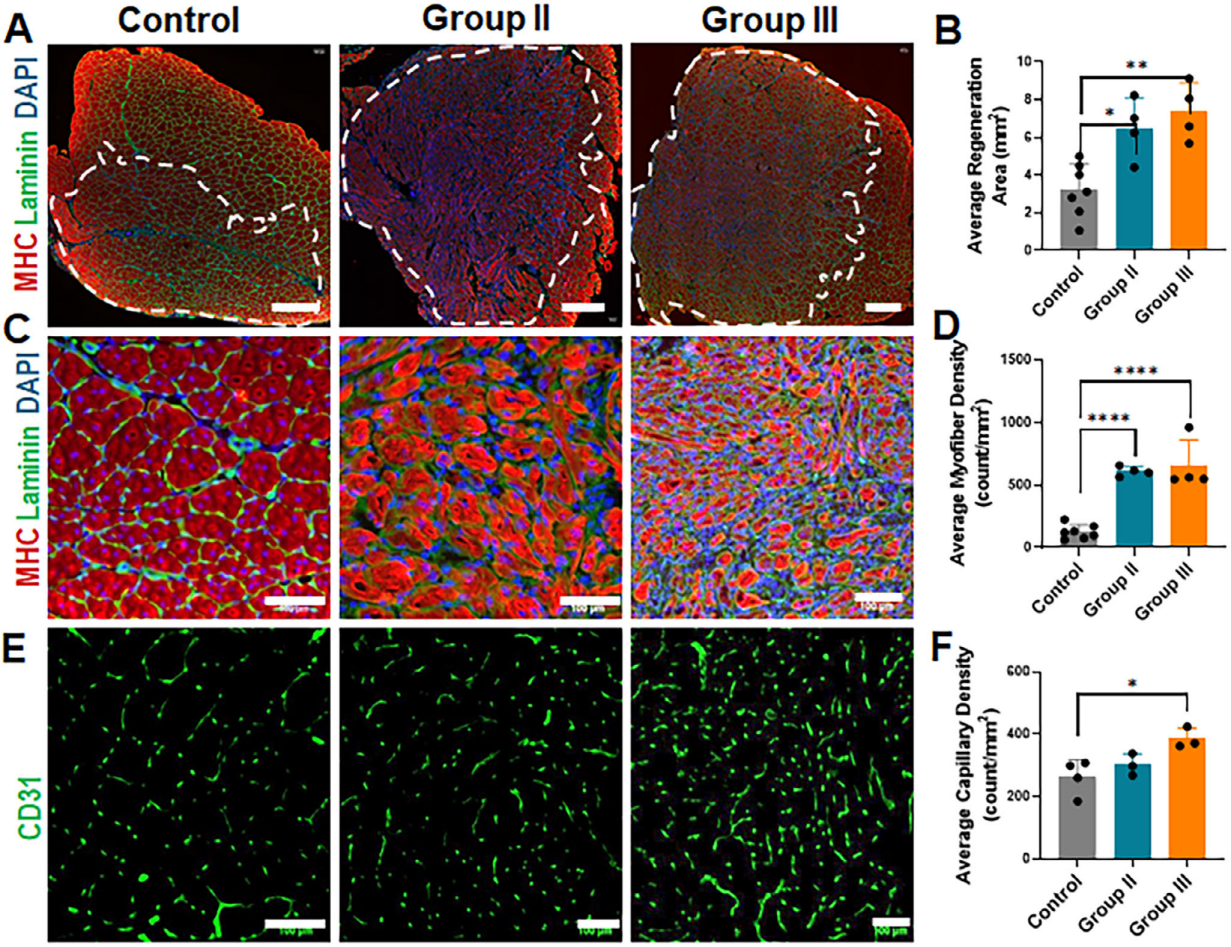
Quantification of muscle and vascular regeneration at 3 weeks post‐implantation of a murine modular muscle unit formed from a rectangular‐shaped mold. Regenerated myofibers are denoted by the expression of MHC, with laminin staining surrounding the border of myofibers. A) Regeneration area as designated by the expression of MHC with centrally located nuclei. Dotted line denotes region of regeneration. B) Quantification of regeneration area (*p = 0.01, **p = 0.002, *n* = 4–7). C) Higher magnification images show muscle regeneration based on myofibers with centrally located nuclei. D) Quantification of myofiber density (****p<0.0001, *n* = 4–7). E) Capillaries within the regenerated muscle based on CD31 expression. F) Quantification of capillary density (*p = 0.002, *n* = 3–4). Scale bars: A (500 µm); C, E (100 µm). Data shown as mean ± SD.

To test the in vivo myogenic potential of C2C12‐derived prefabricated tissues formed with 2 days (Group II) or 4 days (Group III) of pre‐differentiation, the tissue constructs formed from rectangular‐solid shapes were transplanted by aspiration‐assisted delivery (Figure S3, Supporting Information) into the ablated mouse tibialis anterior muscle for assessment of muscle regeneration after 21 days. The effect of 2D pre‐differentiation (2 days vs. 4 days) on the regenerative capacity of biofabricated 3D skeletal muscle tissues was then compared to the negative control group that did not receive any treatment. Immunofluorescence staining and quantification of newly regenerated muscle fibers was performed, based on the expression of myosin heavy chain (MHC) with centrally located nuclei and laminin expression bordering the myofibers (**Figure** **4**A–D). Based on the immunofluorescence images of MHC expression, the myofiber density and area of regeneration were histologically assessed as indicators of muscle regeneration. The average area of regeneration, as defined as the area characterized by MHC expression with centrally located nuclei, was significantly higher for Group II (6.5 ± 1.6 mm^2^, *p = 0.01) and Group III (7.4 ± 1.5 mm^2^, **p = 0.002), in comparison to the negative control group (3.2 ± 1.4 mm^2^), suggesting that pre‐differentiated tissue constructs supported muscle regeneration (Figure 4A,B). The average myofiber density in the pre‐differentiation Group II (608.5 ± 37.8 /mm^2^, ****p<0.0001) and Group III (653.9 ± 204.2 /mm^2^, ****p<0.0001) were significantly higher than the control Group I (125.7 ± 56.5 /mm^2^, Figure 4C,D). Based on the aspect ratio of myofibers expressing MHC and laminin within the regeneration area, the orientation of the newly formed myofibers was 94.00 ± 3.81% cross‐sectional for Group II and 96.09 ± 3.23% for Group III, which was similar to that of the negative control group (97.26%), suggesting proper myofiber organization (Figure S4, Supporting Information). Additionally, the density of CD31 expression, which is a measure of vascular regeneration that is important for sustained muscle regeneration, also showed a statistically significant difference (*p = 0.002) between Group III (385.8 ± 33.4 /mm^2^) and Group I (263.1 ± 56.0 /mm^2^, Figure 4E,F). Together, these data demonstrate that the murine modular tissues could enhance muscle and vascular regeneration in the setting of VML.

**Figure 5:**
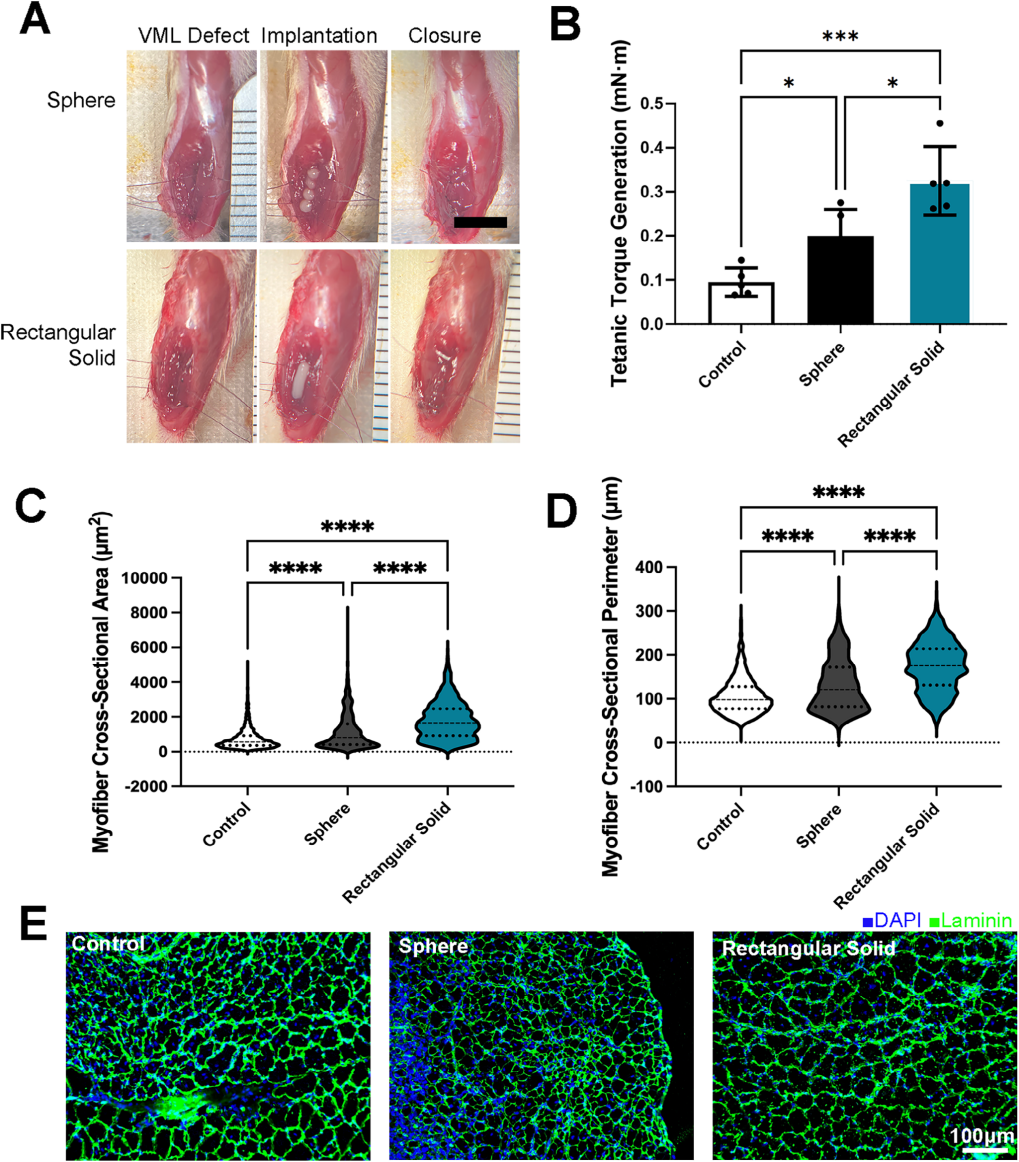
Functional and histological evaluation of regenerated muscle following implantation of scaffold‐free tissue constructs. A) Representative intraoperative images showing VML defect creation, implantation of scaffold‐free tissue constructs of sphere or rectangular solid geometry, and wound closure. Scale bar, 5 mm. B) Quantification of muscle function recovery at 21 days post‐implantation (*p<0.05, ***p = 0.002, *n* = 5). C,D) Quantification of myofiber cross‐sectional area (C) and cross‐sectional perimeter (D) in regenerated tissue (****p<0.0001, *n* = 3). E) Representative immunofluorescence images of regenerated muscle stained for laminin (green) and DAPI (blue). Scale bar, 100 µm. Data shown as mean ± SD.

To further reveal how the role of bioconstruct geometry on muscle function recovery and regeneration, we compared the therapeutic efficacy of C2C12‐derived biofabricated tissues formed with 2 days (Group II) of pre‐differentiation that were shaped into either a single rectangular solid or4 spherical solids. The rationale for choosing spherical bioconstructs was that such a shape does not conform geometrically as well to the rectangular solid‐shaped muscle defect. Using the same total number of starting cells, these two different geometries of muscle tissue units were implanted and compared to the negative control group (**Figure** **5**A). After 21 days, muscle physiology assessment (Figure 5B) revealed a significantly higher mean tetanic torque generation in the group implanted with rectangular solid‐shaped bioconstructs (0.32 ±0.08 mN‐m), compared to the group treated with spheres (0.20 ± 0.06 mN‐m, *p = 0.0164, *n* = 5) or the negative control group (0.10 ± 0.03 mN‐m, ***p = 0.002, *n* = 5), thereby demonstrating that the rectangular solid bioconstruct was more effective than the spherical bioconstruct in muscle function recovery. The group treated with spheres had a significant increase in functional recovery, compared to the negative control group (*p = 0.043, *n* = 5). To further examine the role of bioconstruct geometry on muscle regeneration, we further performed histological analysis of myofiber cross‐sectional area and perimeter analysis within the region of regeneration (Figure 5C–E). The mean myofiber cross‐sectional area was significantly higher in the group treated with the rectangular solid bioconstruct (1790 ± 1066 µm^2^), compared to the group treated with the spherical bioconstructs (1164 ± 1010 µm^2^, *p*<0.0001, *n* = 3)) and the untreated control group (734 ± 597 µm^2^, ****p<0.0001, *n* = 3). Perimeter analysis showed a similar result in which the mean myofiber perimeter was significantly higher in the group treated with the rectangular solid bioconstruct (174 ± 56 µm, *n* = 3), compared to the group treated with the spherical bioconstructs (132 ± 59 µm, ****p<0.0001, *n* = 3) and the untreated control group (107 ± 41 µm, ****p<0.0001, *n* = 3). The spherical bioconstruct group also showed significantly higher myofiber cross‐sectional area and perimeter, compared to the control group (****p<0.0001, *n* = 3). These results indicated that implantation of either bioconstruct geometries improved functional recovery and muscle regeneration over the negative treatment control group, but that the rectangular solid‐shaped geometry was more potent compared to the spherical bioconstructs.

### 2.5 Primary Human Muscle Bioconstructs Support Muscle and Vascular Regeneration, in Part Owing to Pre‐Formed Cell‐Cell Interactions

**Figure 6:**
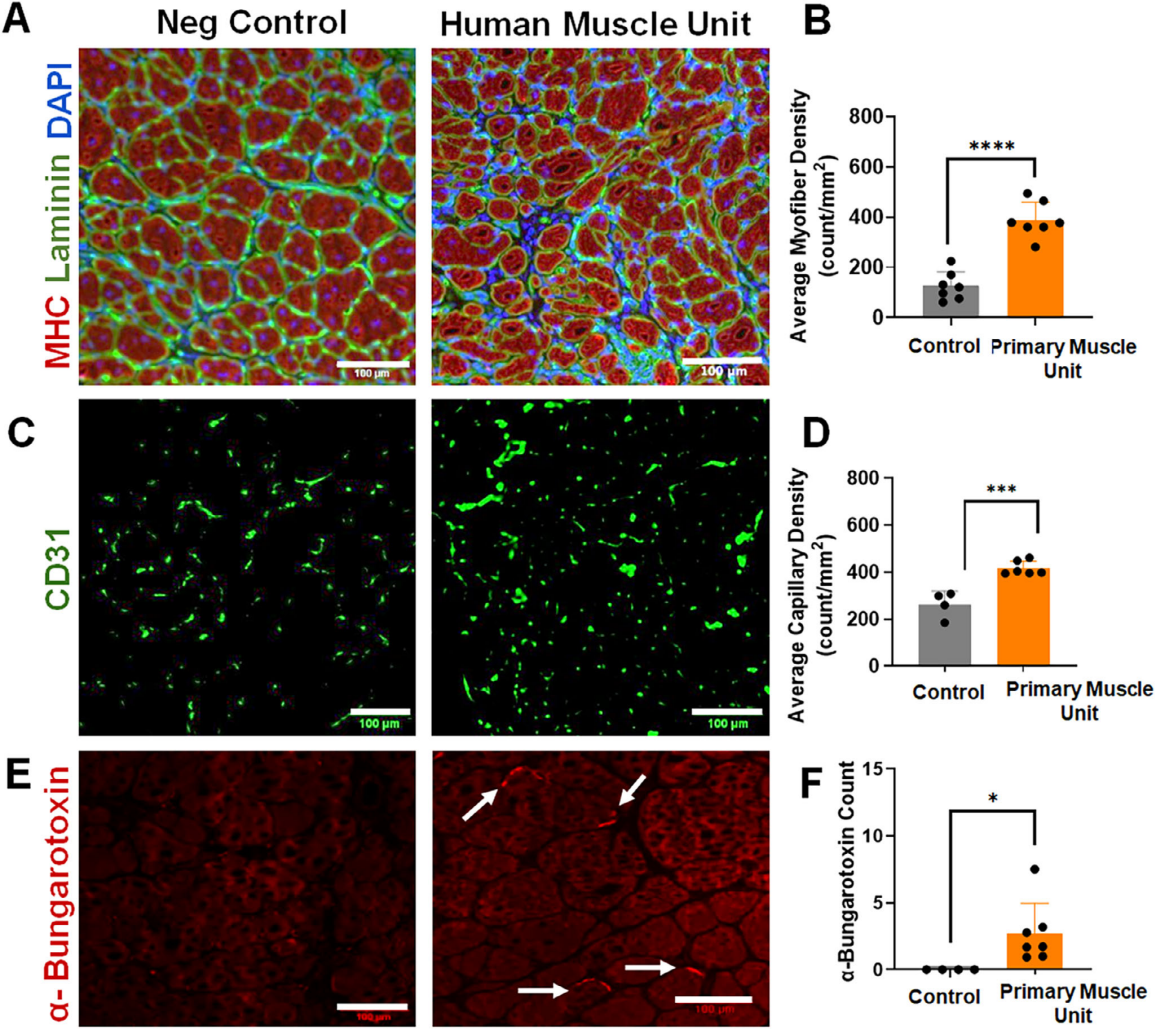
Human muscle units induce muscle and vascular regeneration in a mouse model of VML after 3 weeks. The primary human modular muscle unit was formed from a rectangular‐shaped mold. A) Regenerated myofibers are denoted by the expression of MHC, with laminin staining surrounding the border of myofibers and centrally located nuclei. B) Quantification of myofiber density (****p<0.0001, *n* = 7). C) Capillaries are shown within the regenerated muscle based on CD31 expression. D) Quantification of capillary density (***p<0.001, *n* = 4–6). E) Neuromuscular junctions are visualized by the staining of α‐bungarotoxin. F) Quantification of neuromuscular junctions (*p<0.05, *n* = 4–7). Scale bars: 100 µm. Data shown as mean ± SD.

Having demonstrated the feasibility of implanting modular muscle units derived from C2C12 myoblasts, we next tested the potential of muscle units derived from primary human muscle cells. Using muscle units formed from rectangular solid molds, we aspirated the primary muscle units into the ablated muscle model for 21 days. In comparison to mice without treatment (control), those treated with the primary muscle units were associated with a 3‐fold increase in myofiber density (**Figure** **6**A,B, 388.0 ±70.9 /mm^2^, compared to 127.5 ±56.5 /mm^2^ in the control group, ****p<0.0001), along with a 1.5‐fold increase in capillary density (Figure 6C,D, 417.3 ±30.1 /mm^2^, compared to 263.1 ±56.0 /mm^2^ control, ***p<0.001). Importantly, the muscle treatment group was also characterized by the presence of neuromuscular junctions marked by α‐bungarotoxin staining (2.7 ± 2.3) within the regeneration region that were absent in the negative control group (Figure 6E,F, *p<0.05).

**Figure 7:**
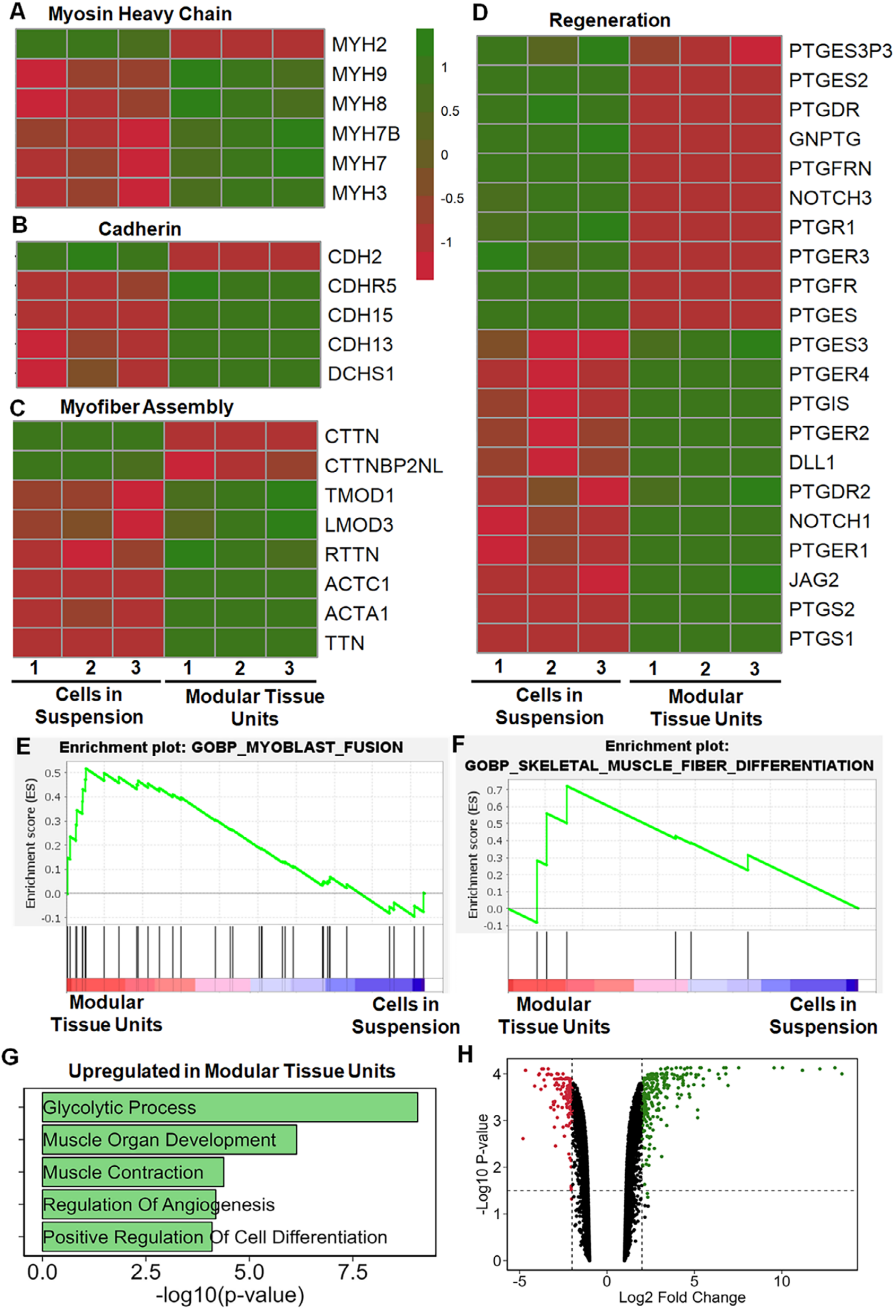
RNAseq data for primary human modular muscle units after 2 days. A–D) Heatmaps of normalized counts for selective genes for cells in suspension and modular tissue units. E,F) Gene set enrichment plot for enrichment of specific gene sets in modular tissue units and cells in suspension. G) Bar plot of gene sets enriched in upregulated genes in modular tissue units in the GO biological process database. H) Volcano plot of fold change (*x*‐axis) and p‐value (*y*‐axis) of genes. Green: Upregulated (log2FC>2) in Modular tissue units, Red: Downregulated (log2FC<‐2) in modular human muscle units (*n* = 3 biological replicates; P_adj_< 0.05).

To better understand how prefabricated scaffold‐free muscle units may be supportive of muscle regeneration, we reasoned that preformed 3D biofabricated scaffold‐free tissues may confer positive cell‐cell interactions that are important for myogenesis and tissue regeneration. Compared to conventional cell delivery modalities in which cells are dissociated and delivered to the target tissue as monodisperse cells, the pre‐formed modular tissue units engage in cell‐cell interactions during the biofabrication process that may have therapeutic advantages. Therefore, RNA Sequencing was performed on primary human tissue constructs that had been pre‐formed within rectangular‐shaped molds for 2 days, compared to primary human muscle myoblasts that were cultured for 2 days in differentiation media while suspended within ultralow adhesion dishes where there was negligible cell‐cell interaction. Transcriptional profiling results show that pre‐formed scaffold‐free tissue units had significantly higher expression (P_adj_<0.05) of multiple skeletal muscle‐specific myosin heavy chains such as *Myh7*, *Myh7b*, and *Myh8* (**Figure** **7**A). Furthermore, consistent with enhanced cell‐cell interactions, the modular tissue units had higher (P_adj_<0.05) expression levels of many cadherins, including *Cdhr5* and *Cdhr13*, demonstrating that modular tissue units conferred enhanced cell‐cell interaction that may contribute to myogenesis (Figure 7B). In addition, myofiber assembly genes such *Tmod1* and *Ttn* were also significantly upregulated (P_adj_<0.05) in the modular tissue, compared to cells cultured in the absence of cell‐cell interactions (Figure 7C). Our results further reveal an upregulation of muscle regeneration pathways such Notch signaling (*Notch1*, *Dll1*, and *Jag1*) and prostaglandin pathway (*Ptgis*, *Ptgs1*, and *Ptgs2*) in the modular units having pre‐formed cell‐cell interactions (Figure 7D). Gene Set Enrichment Analysis (GSEA) was also used to quantify enrichment in gene sets related to Myoblast Fusion and Skeletal Muscle Fiber Differentiation. Both gene sets were significantly enriched in the transcriptome of the modular tissue units (Figure 7E,F, P_adj_<10^−12^). The top 5 gene sets upregulated in the modular tissue units also corresponded to muscle development and functional gene sets (Figure 7G). Finally, the volcano plot highlights that the modular muscle units had notably more upregulated genes than downregulated genes, as well as higher fold change, compared to the cells in suspension (Figure 7H). Some of the highest upregulated genes in modular tissue units correspond to genes such *Lox* (lysyl oxidase) and *Des* (desmin) that are involved in ECM remodeling and myofiber structure, respectively. These results suggest that the transcriptional signature of pre‐formed modular tissue units are primed to support myofiber assembly and regeneration and cell‐cell interactions, compared to monodisperse cells that lack pre‐formed cell‐cell engagement.

### 2.6 Feasibility of Computer‐Assisted In Situ Transplantation of Muscle Bioconstructs

**Figure 8:**
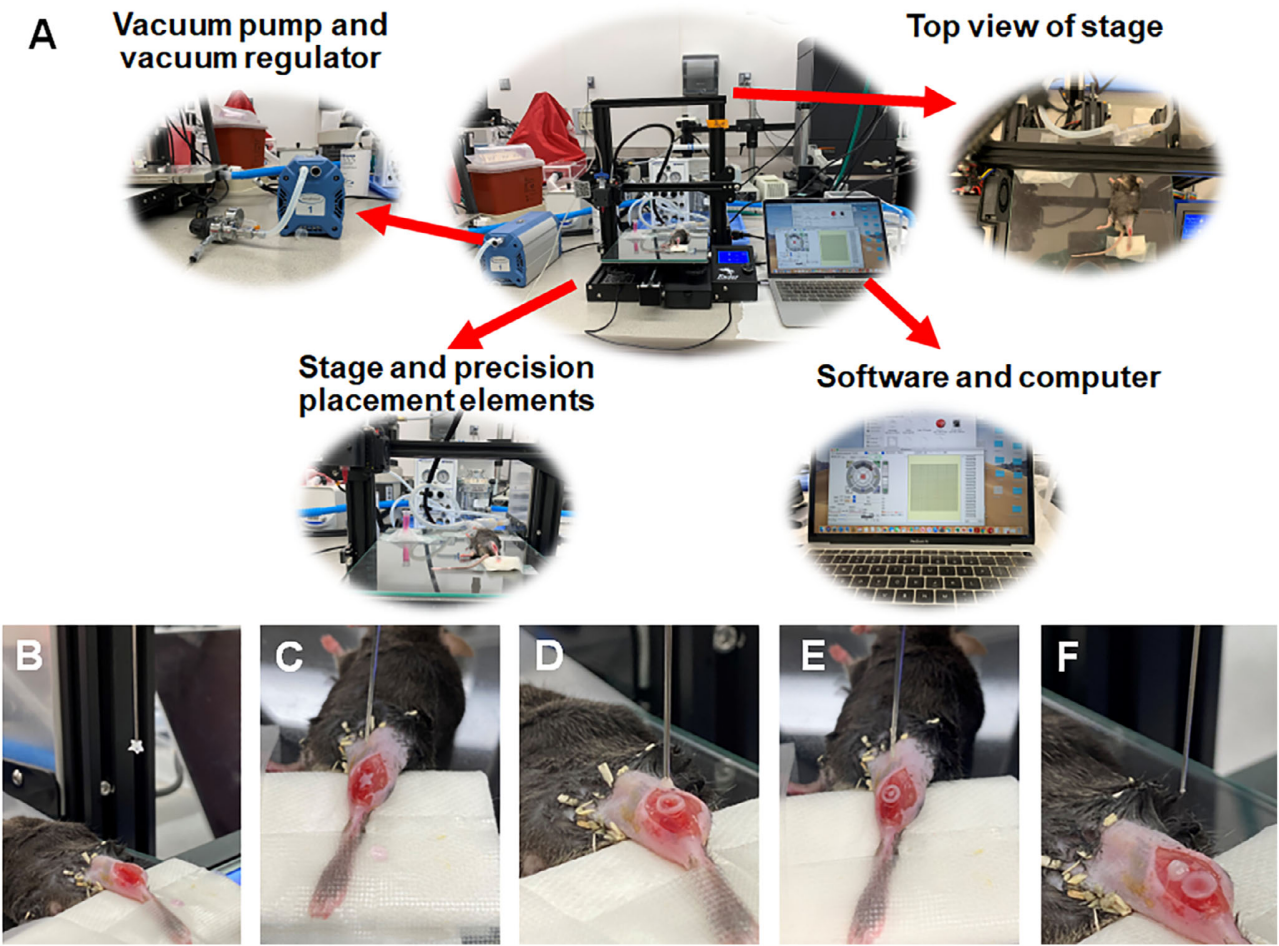
Computer‐assisted in situ muscle tissue implantation into the defect area. A) Components include a vacuum pump and regulator for aspiration, a stage and precision placement elements, and software and computer for precision placement. B,C) A start‐shaped modular muscle tissue was aspirated (B) and then deposited (C) onto the site of the ablated muscle. D,E) In another example, a spheroid‐shaped modular muscle construct was aspirated and then placed into the center of a toroid muscle unit (E). F) A cylindrical muscle unit was placed adjacent to a toroid tissue construct.

Toward clinical translation, to test the feasibility of muscle bioconstructs for intraoperative transplantation, a computer‐assisted automated platform was developed (**Figure** **8**A) using a custom‐fabricated blunt needle and a vacuum pump with regulator that enabled controllable aspiration and deposition of the modular muscle units. The aspiration‐based tissue placement process was monitored and controlled using a computer with graphical user interface (GUI) software, which allowed for the input of custom G‐codes to refine bioprinting parameters. To demonstrate proof‐of‐concept of computer‐assisted intraoperative transplantation, the biofabricated scaffold‐free muscle bioconstructs were programmed to aspirate tissue bioconstructs from a reservoir. The aspiration mechanism enabled the controlled pick‐up and transfer of the tissue constructs, minimizing potential damage or deformation during the printing process. Subsequently, the scaffold‐free tissue constructs were programmed to be deposited by aspiration directly into a VML defect area without utilizing any exogenous biomaterials.

To demonstrate the versatility of the aspiration‐assisted bioconstruct placement approach, various tissue geometries were fabricated using C2C12 myoblasts. For example, star‐shaped (Figure 8B,C) or toroidal (Video S4, Supporting Information) tissue bioconstructs were aspirated and then deposited onto the site of the ablated muscle, demonstrating the system's capability to reproduce complex shapes with intricate features. As an example of automated precision placement, a spheroid shaped modular muscle construct was bioprinted within the center of the toroidal tissue construct (Figure 8D,E, Video S5, Supporting Information). Toward scalability of larger tissues using aspiration‐assisted modular tissue placement, we further show a cylindrical muscle unit placement adjacent to a toroidal tissue construct (Figure 8F, Video S6, Supporting Information). The integration of the custom aspiration‐assisted bioprinting head, coupled with the graphical user interface software and controlled aspiration mechanism, facilitated the accurate and reproducible in situ placement of scaffold‐free tissue constructs directly into the VML defect site. These results highlight that the computer‐assisted aspiration system enables reproducible, controlled placement of prefabricated modular tissue units, which would be challenging to achieve manually with a syringe due to handling limitations and potential tissue deformation.

### 2.7 Toward Clinical Translation—From Medical Imaging to Custom Geometric Design

Toward clinical translation of geometrically tunable muscle bioconstructs as a future technology for personalized muscle repair in the operating room setting, we demonstrated the feasibility of a forward‐thinking pipeline that initiates with the acquisition of high‐resolution, 3D computed tomography (CT) scans of the muscle loss defect for mapping the defect geometry, as is shown in a pilot murine VML defect (Figure S5A,B, Supporting Information). The detailed spatial map, along with computer‐aided design (CAD) software and artificial intelligence (AI), can be employed to translate the imaging information into a digital blueprint, meticulously specifying the precise architecture and dimensions required for the muscle construct (Figure S5C,D, Supporting Information). Using the digital blueprint, along with integrated hardware and shape tunable tissue units, we envision that clinicians will be able to transplant and assemble modular geometrically tunable bioconstructs that assemble together to confirm to the complex wound geometry in the operating room (Figure S5D, Supporting Information), leading to a scalable and personalized muscle tissue regeneration strategy.

## 3 Discussion

The salient findings of this work are that we 1) developed a facile technology to engineer geometrically customizable scaffold‐free muscle tissue constructs (Figure 1) with integration capability (Figure 2); 2) demonstrated the therapeutic benefit of modular scaffold‐free muscle bioconstruct transplantation to augment muscle regeneration (Figures 4, 5, 6); 3) advanced fundamental knowledge of the molecular underpinnings and transcriptional signature by which scaffold‐free muscle units support myogenesis and cell‐cell‐interactions (Figure 3 and Figure 7); and 4) provided a forward‐thinking pipeline of future computer‐assisted intraoperative tissue transplantation for clinical translation (Figure 8; Figure S6, Supporting Information).

The modular tissue approach with aspiration‐assisted placement extends these advantages by directly positioning prefabricated tissues or assembling tissue units onto native tissues or defect sites, facilitating control over the placement and orientation of the biofabricated tissues, thereby potentially enhancing their integration and functional regeneration within the targeted muscle defect area. Our results reveal an important role of bioconstruct geometry in the context of anisotropic tissues such as skeletal muscle. The rectangular solid‐shaped bioconstruct led to significantly higher muscle function recovery, compared to the spherical bioconstruct, along with larger myofiber size (Figure 5). The newly formed myofibers derived from rectangular solid bioconstructs were properly organized along the myofiber direction of the tibialis anterior muscle (Figure S4, Supporting Information). These findings suggest that tissue anisotropy should be considered in the design and assembly of tissue bioconstructs for transplantation. In addition, cell viability analysis further demonstrated that the modular tissues were highly viable post‐fabrication (Figure S2, Supporting Information). This may be a result of the gentle tissue compaction process and secretion of endogenous ECMs that promote tissue stability, which is associated with other forms of scaffold‐free bioconstructs.^[^ ^25^ ^]^ Broadly, these results highlight the potential universality of scaffold‐free modular tissue deployment, as the geometric flexibility and high cellular density of these constructs could support applications across multiple muscle types, injury scales, and even species, thereby strengthening the translational relevance of this approach.

Besides conferring cell‐cell interactions, scaffold‐free muscle bioconstructs are advantageous for clinical translation because they obviate interactions with exogenous ECMs or scaffold degradation byproducts that can induce immune responses. However, clinical translation of scaffold‐free bioconstructs remains in nascent stages, with limited direct in vivo applications reported.^[^ ^26^ ^]^ By contrast, in situ bio‐ink deposition for wound repair and regeneration has progressed further along the translational pipeline, with several studies advancing to animal models and early‐phase clinical trials.^[^ ^27^, ^28^, ^29^, ^30^ ^]^ The approach presented in this study builds upon these existing methodologies by integrating aspiration‐assisted bioconstruct placement with geometric tunability necessary for large‐scale tissue biofabrication. This integration represents a significant step toward the clinical translation of engineered tissues for regenerative medicine and transplantation applications.

The computer‐assisted bioconstruct placement system, in contrast to a manually operated aspiration syringe, provides several potential advantages. First, it enables precise control over placement, orientation, and spacing of modular tissue units, thereby ensuring reproducibility across multiple defects and animals. Second, aspiration‐based retrieval selectively captures the tissue construct without aspirating excess liquid, which improves positional accuracy and stability during deposition. By contrast, manual pipetting typically retains residual liquid around the tissue, which can hinder accurate positioning and compromise reproducibility. Finally, the computer‐assisted system can follow pre‐programmed paths for optimized defect coverage, reducing variability and minimizing potential human error. Although we did not perform a formal experimental comparison with manual deposition, the accuracy and reproducibility observed in our pilot experiments underscore the benefit of integrating computer assistance into the aspiration‐assisted deposition workflow.

Aspiration‐assisted placement of engineered tissues is an emergent bioprinting technique that leverages aspiration forces to manipulate and position scaffold‐free tissues with high spatial accuracy, maintaining high cell viability and structural fidelity both in 2D and 3D. Aspiration based placement of engineered tissues can be useful for many applications in regenerative medicine. As shown in this and other published work, aspiration‐assisted placement can be applied toward biofabricating various tissue models, including bone,^[^ ^31^ ^]^ cartilage,^[^ ^32^ ^]^ osteochondral interfaces,^[^ ^33^ ^]^ cardiac,^[^ ^34^ ^]^ and breast cancer models.^[^ ^35^ ^]^ Among the existing tissue placement techniques include Kenzan, which utilizes a needle array for controlled spheroid placemen,^[^ ^25^ ^]^ and a high‐throughput version of aspiration‐assisted bioprinting which employs a nozzle array to simultaneously position multiple same‐size spheroids.^[^ ^26^ ^]^ These methods offer a high degree of spatial accuracy and reproducibility, making them well‐suited for the fabrication of complex, pre‐designed tissue constructs for applications in regenerative medicine, drug screening, and disease modeling. However, these previously reported techniques require uniformly sized spheroids to build larger tissue constructs. In contrast to published work, our prefabrication approach enables the assembly of relatively modular tissue units in diverse geometries, thereby allowing for greater flexibility in tissue shape, enhanced structural integrity, and improved scalability for clinical applications. This approach may be broadly applicable across different muscle types, injury scales, and species, and could be adapted for human translation.

Geometrically tunable muscle bioconstructs provide a foundation for future intraoperative tissue reconstruction strategies, enabling complex geometries and biomimetic architectures to enhance tissue integration and regeneration. The ability to fabricate custom geometries expands the possibilities for recreating biomimetic tissue architectures that more closely resemble native tissue structures. However, the ability to manufacture autologous or allogenic sources of scaffold‐free modular units for intraoperative use will need to be addressed before clinical translation. Furthermore, future work is required to evaluate long‐term stability, integration, immune compatibility, and scalability of modular tissue assembly for larger defects.

While our current study demonstrates tissue integration and function over a 21‐day period in a small‐animal model, longer‐term time points would be beneficial for in vivo studies and transcriptomic profiling to evaluate the stability, host integration, and sustained neuromuscular functionality of these scaffold‐free modular muscle units. Genetic gain‐ and loss‐of‐function studies will further aid in identifying key signaling pathways that underlie myogenesis within pre‐formed scaffold‐free tissue bioconstructs. Moreover, although the computer‐assisted deposition workflow allows precise positioning, scaling for larger defects or clinical use will require further development of hardware, software, and operational protocols. These considerations emphasize both the potential and current limitations of modular tissue implantation pipelines for potential intraoperative applications.

Clinically, modular scaffold‐free tissue deployment holds promise for personalized regenerative medicines with designing personalized constructs and using autologous cell resources. A clinical advantage of the modular tissue approach is that it eliminates the need for prefabricated scaffolds or constructs, while enabling the scalability to meet the demands of larger muscle defects. Furthermore, modular tissue bioconstructs obviate donor site morbidity associated with autologous muscle flaps, which is the surgical gold standard. We envision that surgeons in the future will utilize a bioprinter equipped with an aspiration head and a dispensing nozzle. The aspiration head gently retrieves modular muscle tissues from a reservoir, analogous to a specialized tool collecting building blocks comprising various cell types. The dispensing nozzle then meticulously deposits various modular tissue units comprising muscle, vascular, and nerve bioconstructs in a layer‐by‐layer fashion, resembling the assembly of a complex 3D model. This analogy aptly captures the essence of the technique, where each deposited layer represents a specific section of the muscle, meticulously designed to mimic the intricate architecture. The inclusion of AI will further streamline the intraoperative pipeline by facilitating seamless integration of the bioprinted tissue with the surrounding healthy muscle tissue. By analyzing the CT scans and the generated CAD file, AI algorithms may provide recommendations for optimal bioprinting strategies using modular tissue units. These recommendations may encompass the selection and formulation of tissue building blocks, the printing pattern necessary to recapitulate specific muscle structures, and the identification of potential challenges that might arise during the bioprinting process. The workflow can be further adapted for on‐demand use through libraries of pre‐made modular bioconstructs comprising various geometries and sizes, sourced from allogeneic cells or those derived from human induced pluripotent stem cells, for intraoperative assembly to fill the muscle defect. For intraoperative use, the pipeline can be further revised to semi‐automated or surgeon‐assisted deposition, potentially allowing intraoperative modular tissue assembly to be performed without highly specialized equipment, while still maintaining positional accuracy and reproducibility, thereby increasing their universal utility. These considerations emphasize both the potential and current limitations of computer‐assisted intraoperative tissue placement.

## 4 Conclusion

In conclusion, we demonstrate a facile technology to engineer scaffold‐free high‐density muscle bioconstructs with customizable shapes and sizes for muscle regeneration applications. The scaffold‐free environment of the tissue units fostered cell–cell interactions that supported myogenesis, as demonstrated by transcriptomic profiling. Rectangular solid‐shaped muscle bioconstructs were transplanted into the site of the defect in a mouse VML model, where the muscle bioconstruct promoted muscle functional improvement and induced muscle and vascular regeneration after 21 days. By combining high‐cell density, geometric tunability, structural integration, and transcriptional priming to support functional regeneration, this scaffold‐free tissue engineering strategy represents a promising approach for treatment of VML with clinical translation potential.

## 5 Experimental Section

### Cell Culture

Mouse myoblast (C2C12, cat #CRL‐1772, adult female donor, spontaneous immortalization, ATCC) and HMEC (cat # CRL‐3243, neonatal male donor, immortalized by SV40 large T antigen insertion, ATCC) cell lines, along with primary human skeletal muscle myoblast (cat # A12555, mycoplasma pre‐screened, Gibco), were cultured in growth medium consisting of Dulbecco's modified Eagle's medium (DMEM) with 20% fetal bovine serum (FBS) and 1% penicillin/streptomycin. For myoblast differentiation studies, differentiation media composed of DMEM containing 2% horse serum and 1% penicillin/streptomycin medium was utilized to induce differentiation and fusion of myoblasts. All cells were maintained at 37 °C and 5% CO_2_ humidified atmosphere. The cell culture medium was changed every other day. The C2C12 and HMEC immortalized cell lines were used at passages 5 through 24, whereas primary human myoblasts were used within two passages. Where indicated, C2C12 cells were fluorescently labeled with GFP^[^ ^19^ ^]^ to facilitate fluorescence imaging. Similarly, in specified experiments, HMEC cells were fluorescently tagged with mRuby fluorescent reporter gene, according to the previously studies.^[^ ^19^ ^]^

### Fabrication of Scaffold‐Free Modular Tissues

A facile approach to use fabricated molds to guide the shape of the scaffold‐free modular tissue units was developed. (Figure S1, Supporting Information). Various 3D shapes depicting a star, sphere, hexagon, and different alphabet letter shapes were designed in Solidworks (Waltham, MA) and then 3D printed with an Ender 3 printer (Creality) using 1.75 mm Polylactic acid (PLA) filaments (Hatchbox 3). The printed shapes were custom‐designed within a specified range in sizes from 0.2 to 6 mm. The shapes were placed in the center of a 12‐well plate (Figure S1A, Supporting Information) before 2 ml of 1% (w/v) agarose solution was poured into the well and were kept at 4 °C for 30 min (Figure S1B, Supporting Information). The cast agarose samples were removed from the well plate using a round‐end spatula (Figure S1C, Supporting Information). A tweezer was used to remove the PLA shapes from the agarose, leaving behind a mold resembling the PLA shape (Figure S1D, Supporting Information). The molds were then transferred into new 6‐well plates (Figure S1E, Supporting Information). Before seeding, the agarose molds were disinfected with 70% ethanol and then washed with cell medium three times.

Subconfluent cultures of C2C12 myoblasts, HMEC endothelial cells, or primary skeletal muscle cells were dissociated using a TrypLE Express Enzyme (Thermofisher) and then reconstituted, at a concentration where specified at 1–4 × 10^6^ cells in 100 µL of growth medium and then pipetted into each agarose mold (Figure S1F, Supporting Information). After 15 min of incubation within the mold at 37 °C, 10 mL of differentiation medium was added per well (Figure S1G, Supporting Information). The medium was changed every other day for up to 5 days. Scaffold‐free tissue formation was monitored daily on a fluorescence microscope system (Keyence). Where specified, co‐cultures of C2C12 and HMEC cells were seed into the molds at a 1:1 ratio with a total specified cell density of 1‐4 × 10^6^ cells per mold. At indicated timepoints, the tissue bioconstructs were aspirated for in vitro analysis (Figure S1H, Supporting Information). For in vivo implantation, the tissue constructs were placed using an aspiration needle into a reservoir consisting of differentiation media for later aspiration into the muscle defect (Figure S1I, Supporting Information).

### Cell Viability of Modular Scaffold‐Free Bioconstructs

To assess the cell viability of rectangular solid‐shaped bioconstructs, C2C12 myoblasts (2 × 10^6^ cells per bioconstruct) were seeded into the agarose molds. After 2 days, the bioconstructs were incubated with vital dyes comprising 1 µM calcein AM and 1.6 µM ethidium homodimer‐1 (Life Technologies) in phosphate buffered saline (PBS) for 45 min. The bioconstructs were then washed with PBS twice before imaging in a confocal laser scanning microscopy (LSM710, Zeiss, Germany) at 20 µm increments within the z‐stacks. For comparative cell viability analysis among varying geometric shapes, 2 × 10^6^ GFP‐labeled C2C12 myoblasts were cultured in agarose molds of varying shapes comprising equivalent shape volumes for 2 days in growth media. Ethidium homodimer‐1, which labels non‐viable cells, was incubated within the constructs. Z‐stacks were acquired on a Nikon A1 HD25 confocal microscope (10 µm steps, total depth 300 µm) using identical settings. 3D reconstruction of the images was generated in Imaris (v9.0). Image stacks were analyzed using Python and Cellpose (Segment Anything model, Cellpose v4.0.6), which segmented GFP‐positive and Ethidium homodimer‐1‐positive cells per slice. Cell viability as a function of depth was performed by quantifying the percentage of non‐viable cells based on positive expression of ethidium homodimer‐1 expressed as a percentage of total cells (*n* = 3). Cells that were not permeable to ethidium homodimer‐1 were assumed to be viable cells.

### Integration of Modular C2C12 and HMEC Tissue Units In Vitro

To demonstrate the feasibility of fusion among adjacent modular tissues, L‐shaped PLA structures were designed using computer‐aided design (CAD) software and then 3D‐printed (Prusa MK4 3D Printer). The L shapes (≈6 × 6 × 2 mm) were sterilized and filled with 2% agarose and then allowed to solidify at room temperature. Once solidified, the shapes were removed, resulting in uniform agarose molds with L‐shaped cavities. GFP‐labeled C2C12 myoblasts (4 × 10^6^ cells) or mRuby‐labeled HMECs (4 ×10^6^ cells) were seeded into L‐shaped molds, with additional 2 million cells added one day later to account for cell contraction in the molds. After 3 days, the L‐shaped tissues were transferred into a new agarose mold containing a removable separator to maintain initial separation of the tissue blocks. Another 2 days later, the separator was removed, allowing direct contact between the modular tissue blocks. The tissues were incubated for 3 additional days to facilitate fusion among the adjacent modular units (*n* = 3 samples). High‐resolution confocal imaging (Nikon A1R‐Si HD confocal microscope) was performed to evaluate the fusion between GFP‐tagged C2C12 (green‐colored) and mRuby‐tagged HMEC (red‐colored) tissue blocks after 3 days of direct cell‐cell contact at the interface. The Z‐stack images were captured at 10x and 20x magnification, with settings optimized to reduce photobleaching while ensuring clear resolution of the fusion interface.

Integrated fluorescence intensity analysis of GFP and mRuby signals (*n* = 3 samples) in the Z‐stack images was performed to evaluate spatial distribution and cellular infiltration of C2C12 and HMEC populations at the interface between adjacent shapes. For fused tissues, three spatial regions were defined for quantitative analysis: the in situ region, representing the central location of the tissue module; the proximal region, represented by the region adjacent to the tissue construct; and the distal region, at a location which is > 2 mm away from the interface of the two tissue modules. The integrated fluorescence intensity of GFP and mRuby signals was measured within each defined region. Subsequently, quantitative analysis of mean fluorescence intensities was compared between single modular tissues and the defined spatial regions (in situ, proximal, distal) of the fused tissues, in order to evaluate cellular infiltration across the fusion interface. Fluorescence images were processed and analyzed using ImageJ 1.54f (National Institutes of Health, USA) to quantify integrated fluorescence intensity (*n* = 3).

### RNA Sequencing

RNA Sequencing was performed on modular muscle bioconstructs formed from rectangular solid molds that were composed of either murine C2C12 myoblasts or primary human muscle cells. For the murine muscle tissue units formed from C2C12 myoblasts, the experimental groups consisted of muscle units formed from cells with pre‐differentiation treatment as were denoted as follows: **Group I**: 2 million C2C12 myoblasts were seeded into molds on day 0 without prior differentiation and then maintained in the mold in differentiation media for 5 days. **Group II**: C2C12 cells were subjected to 2 days of pre‐differentiation on tissue culture plastic before seeding in the mold (2 million cells per mold) for an additional 3 days. **Group III**: C2C12 cells were subjected to 4 days of pre‐differentiation on tissue culture plastic before seeding to the molds (2 million cells per mold) for an additional 1 day (*n* = 4–5 per group).

In addition, to study the molecular effects by which pre‐formed cellular interactions present within the modular muscle units may prime muscle myogenesis after implantation, RNA Sequencing was performed on human muscle units composed of 2 million primary human muscle cells cultured within rectangular solid‐shaped molds for 2 days in growth media prior to RNA isolation. As a basis for comparing to pre‐formed inter‐cellular interactions within the modular unit in the setting of VML, an additional group consisting of monodisperse primary human muscle cells suspended in ultralow adhesion dishes (Corning) for 2 days was included, in which there was negligible cell‐cell interaction in suspension (*n* = 3 per group).

Total RNA of each sample was isolated using the GeneJET RNA Isolation Kit (Fisher Scientific). The samples underwent library preparation and bulk RNA sequencing by Novogene Corporation, as done previously.^[^ ^36^, ^37^ ^]^ In brief, poly‐T oligo‐attached magnetic beads were used to purify messenger RNA, followed by fragmentation and cDNA synthesis using dUTP for directional library preparation. High‐throughput sequencing using the Novaseq 6000 was used for sequencing, and raw data was created using CASAVA base recognition in FASTQ format files. The files contained the sequences of reads and corresponding base quality. Data quality control was performed by quantifying the error rate in reading using adapter sequences and GC content. Alignment of sequenced reads to reference human/mouse genome was performed using HISAT2. Gene expression levels in form of FPKM (fragments per kilobase of transcript sequence per million base pair sequenced) was obtained for each unique gene.

Downstream data analysis using the counts was done by normalizing the FPKM counts and log transforming the data. Differential expressed genes (DEGs) were obtained by the lmFit function of package limma in R. Specific comparisons between groups were statistically tested in the model using the contrasts fit function. The adjusted P value (P_adj_) and fold change were calculated for each comparison, where significance was accepted at P_adj_<0.05. Specific differential gene expression lists were made for specific comparisons to enable enrichment analysis on EnrichR website. For GSEA analysis, the java desktop application was used, with instructions from the Wiki page. The bar plots were made using ggplot package and the pheatmap package was used to create all the heatmaps in the figures.

### Therapeutic Efficacy of Scaffold‐Free Modular Muscle for Treatment of VML

To study the therapeutic benefit of prefabricated scaffold‐free tissues on muscle regeneration, it was next implanted a scaffold‐free muscle unit derived from a rectangular solid mold or four detached spheres into the void space of the mouse tibialis anterior muscle after induction of VML. In these experiments, the prefabricated tissue constructs were aspirated using custom aspiration bioprinting heads using negative pressure to manually transfer the tissue construct from the holding reservoir into the ablated muscle region (Figure S3, Supporting Information). The muscle unit was formed by C2C12 myoblasts (2 million cells) that were cultured in differentiation media in tissue culture polystyrene dishes for 2 or 4 days, followed by subsequent culture within a single rectangular solid‐shaped mold (≈ 5 × 1.5 × 1 mm) or 4 spherical‐shaped molds (1.25 mm diameter) for a total of 5 days prior to implantation (*n* = 4–7 per group). The control group consisted of animals with VML induction but no treatment. To examine the therapeutic efficacy using clinically relevant primary human muscle cells, a second study was performed in which animals received tissue constructs composed of 2 million primary human muscle cells expanded in growth media within rectangular solid‐shaped molds for 2 days (*n* = 3–7 per group).

VML was induced in immunodeficient (NOD.Cg‐Prkdc scid Il2rg tm1Wjl /SzJ) mice (8–9 weeks old, male, Jackson Laboratory) by first anesthetizing and maintaining with 1%–3% isoflurane and an oxygen flow rate of 1 L min^−1^. A skin incision was made to expose the tibialis anterior muscle. Bilateral VML was performed by excising 40% of the tibialis anterior muscle volume, according to the previous studies,^[^ ^19^, ^38^ ^]^ to generate a rectangular solid‐shaped muscle defect (7 × 2 × 3 mm). Mice were randomized to receive a tissue bioconstruct or no transplant (negative control). Tissue bioconstructs were aspirated into the ablated muscle, followed by suture closure of the muscle layer and skin flap. Water and a standard rodent diet were given ad libitum under a standard 12 h light/12 h dark cycle. At the end of the 21‐day study, animals were euthanized by isoflurane induction and thoracotomy, followed by extraction of the tibialis anterior muscle for histological analysis of muscle regeneration. All animal procedures were performed in accordance with the Guidelines for Care and Use of Laboratory Animals of the Veterans Affairs Palo Alto Health Care System and approved by the Institutional Animal Care and Use Committee (HUN1555).

### Muscle Physiology

Where indicated, muscle force generation was assessed 3 weeks after implantation of a rectangular solid‐shaped bioconstruct or spherical bioconstruct, compared to the negative control group. In the anesthetized state, the knee of each mouse was positioned in a brace, with the foot secured to a transducer (3‐in‐1 Whole Animal System for Rat and Mouse, Aurora Scientific, ON, Canada). Two electrodes were inserted subcutaneously into the skin directly above the tibialis anterior muscle.^[^ ^39^ ^]^ Dynamic Muscle Control/Analysis (DMC/DMA) LabBook software suite (Aurora Scientific, ON, Canada) was used to deliver tetanic stimulation of the muscle at 150 Hz and to analyze the resulting data. The same procedure was performed on the contralateral tibialis anterior muscle, and the average tetanic torque from both legs was recorded for analysis (*n* = 5 each group).

### Histological Analysis of Muscle Regeneration, Vascular Regeneration, and Innervation

After 21 days following induction of VML, the animals were euthanized and the tibialis anterior muscles were extracted for cryosectioning. Histological analysis was performed to examine muscle regeneration, vascular regeneration, and neuromuscular junction formation (n = 3‐7 per group) using established methods based on our previous publications.^[^ ^38^, ^40^, ^41^ ^]^ In brief, tissue cross sections were fixed for 15 min in 4% paraformaldehyde (Thermo Scientific), washed three times with PBS, and permeabilized in 0.5% Triton‐X100 (Sigma) for 10 min. Samples were blocked in 1% bovine serum albumin (Sigma) and all subsequent steps were performed using 0.1% bovine serum albumin for antibody dilutions. For assessment of muscle regeneration, fixed samples were incubated with a primary antibody against myosin heavy chain (MHC, cat # M4276, Sigma) overnight at 4 °C followed by Alexa Fluor‐594 (cat # A‐11032, Invitrogen). For co‐staining of laminin or endothelial cells, samples were then incubated with laminin marker (cat # ab11575, Abcam) or CD31 antibody (cat # 550 274, BD Biosciences) overnight at 4 °C followed by Alexa Fluor‐488 antibody (cat #s A‐11006 and A‐11008, Invitrogen). For staining of neuromuscular junction markers, samples were incubated with Alexa Fluor‐594 conjugated α‐bungarotoxin (cat # B13423, Invitrogen). All samples were treated with Fluoroshield with DAPI (Sigma–Aldrich) nuclear stain prior to coverslip placement.

Images were acquired using a fluorescence microscope (BZ‐X810, Zeiss) to capture the entire cross‐section of the tissue and then montaged together. The montaged images were used to quantify area of regeneration based on the region of tissue sample with centrally located nuclei. The myofiber density of regenerating myofibers was defined as the number of MHC‐expressing myofibers with centrally located nuclei, normalized to the area of regeneration (ImageJ software). To calculate capillary density based on the CD31 expression, all capillaries within the area of regeneration were counted using ImageJ software and normalized to the corresponding area of regeneration.^[^ ^38^ ^]^ The number of α‐bungarotoxin‐stained neuromuscular junctions were counted within the regenerative area using ImageJ software.^[^ ^38^, ^41^ ^]^

For quantification of myofiber orientation within tissue cross‐sections, images of myofibers within the region of regeneration were computed for aspect ratio using custom Python code, in which the myofibers labeled by MHC and laminin were segmented for quantification of myofiber aspect ratio. An aspect ratio less than 3 represented cross‐sectional myofibers and an aspect ratio greater than 3 represented longitudinally oriented myofibers. The percentage of myofibers in the cross‐sectional or longitudinal orientations were then expressed as a percentage of total myofibers within the region of regeneration (*n* = 3). For quantification of regenerated myofiber area and perimeter, representative images of myofibers labeled by laminin underwent processing and morphological analysis using Python 3.8.12 with the following key packages: Cellpose v4.0.6 for deep learning‐based segmentation, NumPy v1.24.4 and SciPy v1.10.1 for numerical computations, scikit‐image v0.21.0 for morphological feature extraction, OpenCV v4.5.5 for image preprocessing, and pandas v2.0.3 for data processing. Muscle fiber cross‐sections from the regeneration area were automatically segmented using a dual‐model approach comprising a custom‐trained Cellpose model optimized for muscle fiber morphology and a baseline Cellpose SAM model, both implemented with PyTorch v2.4.1 and TorchVision v0.20.0 for GPU acceleration. Segmented objects were subjected to stringent quality filters to exclude artifacts: minimum area threshold of 100 pixels, maximum area threshold of 5 × 10^4^ pixels, aspect ratio limit of 10, solidity threshold of 0.55, and automatic removal of boundary‐touching objects. Morphological features were extracted for each validated fiber using scikit‐image regionprops analysis, including area, perimeter, major, and minor axis lengths, aspect ratio, circularity, eccentricity, solidity, extent, and equivalent diameter. Muscle myofiber perimeter and area analysis was quantified among a minimum of 1000 myofibers derived from 3 independent samples per group.

### Feasibility of Computer‐Assisted Intraoperative Implantation of Muscle Bioconstructs into Mouse Muscle Defect

Toward clinical translation, the compatibility of modular muscle placement for intraoperative use in conjunction with medical imaging for treatment of VML was tested (Figure S6, Supporting Information). A custom‐made pipette holder was designed in Solidworks and 3D printed using the Ender 3 printer (Creality). A 3D printed pipette holder was inserted as the printer head. In order to generate and maintain the aspiration pressure during bioprinting, a vacuum pump (Vacuubrand) with a regulator (Megasan, Turkiye) was utilized. A 3‐cc syringe barrel (Nordson, RI) with a 27G needle was mounted into the pipette holder and the end of the vacuum regulator was connected to the barrel with a proper tubing. A GUI host (Pronterface) was used to control the motion stage, vacuum, and send G‐code via 3D printer. Animals were placed on the heated stage and VML defects were made. Prior to the bioprinting of tissues, the top of the VML defects was identified and recorded as a bioprinting point in the software interface. Based on this calibration, intraoperative bioconstruct placement was performed with a custom G‐code at a speed of 100 mm s^−1^. Muscle modular tissue units composed of C2C12 myoblasts with the shapes of a disc, toroid, or sphere were collected from the reservoir with backpressure and deposited at the site of VML. To test the feasibility of integrating modular tissue formation with clinical imaging modalities in a pilot animal, it was performed micro computed tomography (Skyscanner, Bruker) imaging of a murine tibialis anterior muscle after induction of VML to visualize the 3D defect geometry. Computer aided design (Fusion 360 software) was then used to map the defect geometry.

### Statistical Analysis

Data were shown as mean ± standard deviation. All statistical analysis was performed using GraphPad PRISM version 10. Where appropriate, a one‐way analysis of variance (ANOVA) was performed with post hoc Tukey's adjustment for three or more groups. Statistical analysis between two groups was performed by a Student's t‐test. Differences were considered significant at *p*<0.05.

### Ethics Approval Statement

All animal procedures were performed in accordance with the Guidelines for Care and Use of Laboratory Animals of the Veterans Affairs Palo Alto Health Care System and approved by the Institutional Animal Care and Use Committee (Protocol HUN1555).

## Conflict of Interest

The authors declare no conflict of interest.

## Supporting information



Supporting Information

Supplemental Video 1

Supplemental Video 2

Supplemental Video 3

Supplemental Video 4

Supplemental Video 5

Supplemental Video 6

## Data Availability

The data that support the findings of this study are available from the corresponding author upon reasonable request.
