# Supplementary material for: Geometrically Tunable Scaffold‐Free Muscle Bioconstructs for Treating Volumetric Muscle Loss
Source: Adv Healthc Mater. 2025 Oct 23;15(9):e01887. doi: 10.1002/adhm.202501887 (PMC12971103; doi:10.1002/adhm.202501887)
Supplement: Supplementary file 1 — Supporting Information [file ADHM-15-0-s004.docx]

**Supporting Information**

**Geometrically Tunable Scaffold-Free Muscle Bioconstructs for Treating Volumetric Muscle Loss**

Bugra Ayan ^1,2#^, Gaoxian Chen^1,2,3#^, Ishita Jain^1,2,3^, Sha Chen^1,2,3^, Gladys Chiang^3^, Caroline Hu^3^, Renato Reyes^3^, Beu P. Oropeza^1,3^, Ngan F. Huang^1,2,3,4*^

^1^ Department of Cardiothoracic Surgery, Stanford University, Stanford, CA, 94305, USA

^2^ Stanford Cardiovascular Institute, Stanford University, Stanford, CA, 94305, USA

^3^ Veterans Affairs Palo Alto Health Care System, Palo Alto, CA, 94304, USA

^4^ Department of Chemical Engineering, Stanford University, Stanford, CA 94305, USA

^#^ Contributed equally

***** Email: ngantina@stanford.edu

**SUPPLEMENTAL FIGURES**


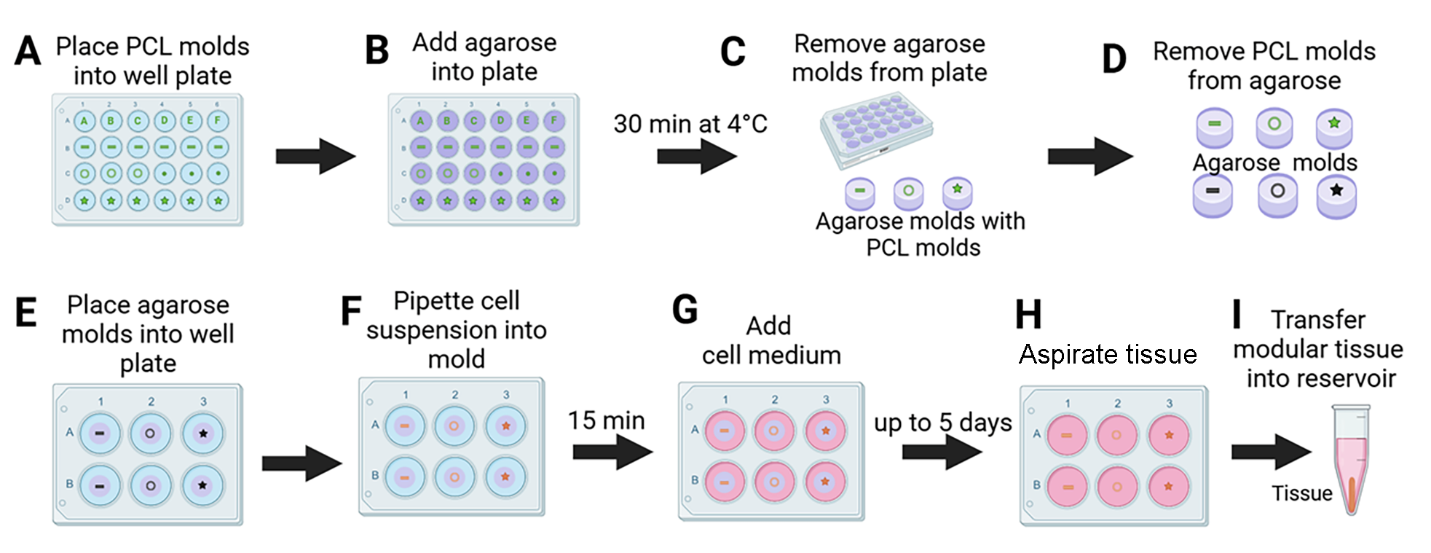


**Figure S1. Schematic depiction of scaffold-free fabrication of modular tissue units. A.** Various shapes are 3D printed using polylactic acid (PLA) and then placed into well dishes. **B.** Agarose is then added to each well to create a mold. **C.** After allowing the agarose to form a gel, the agarose and shapes are removed from the well dish using a spatula. **D.** The PLA shapes are removed with tweezers. **E.** The agarose molds are then placed into new well plates. **F.** Suspension cells are pipetted into the mold. **G.** After 15 min, media is added into the well.  **H.** At various time points, the modular tissues are aspirated for collection. **I.** For in situ or intraoperative bioprinting, the modular tissues are transferred into a reservoir containing cell media for later aspiration into the muscle defect region. Created in BioRender. Huang, N. (2025)

https://BioRender.com/m93h230.

**
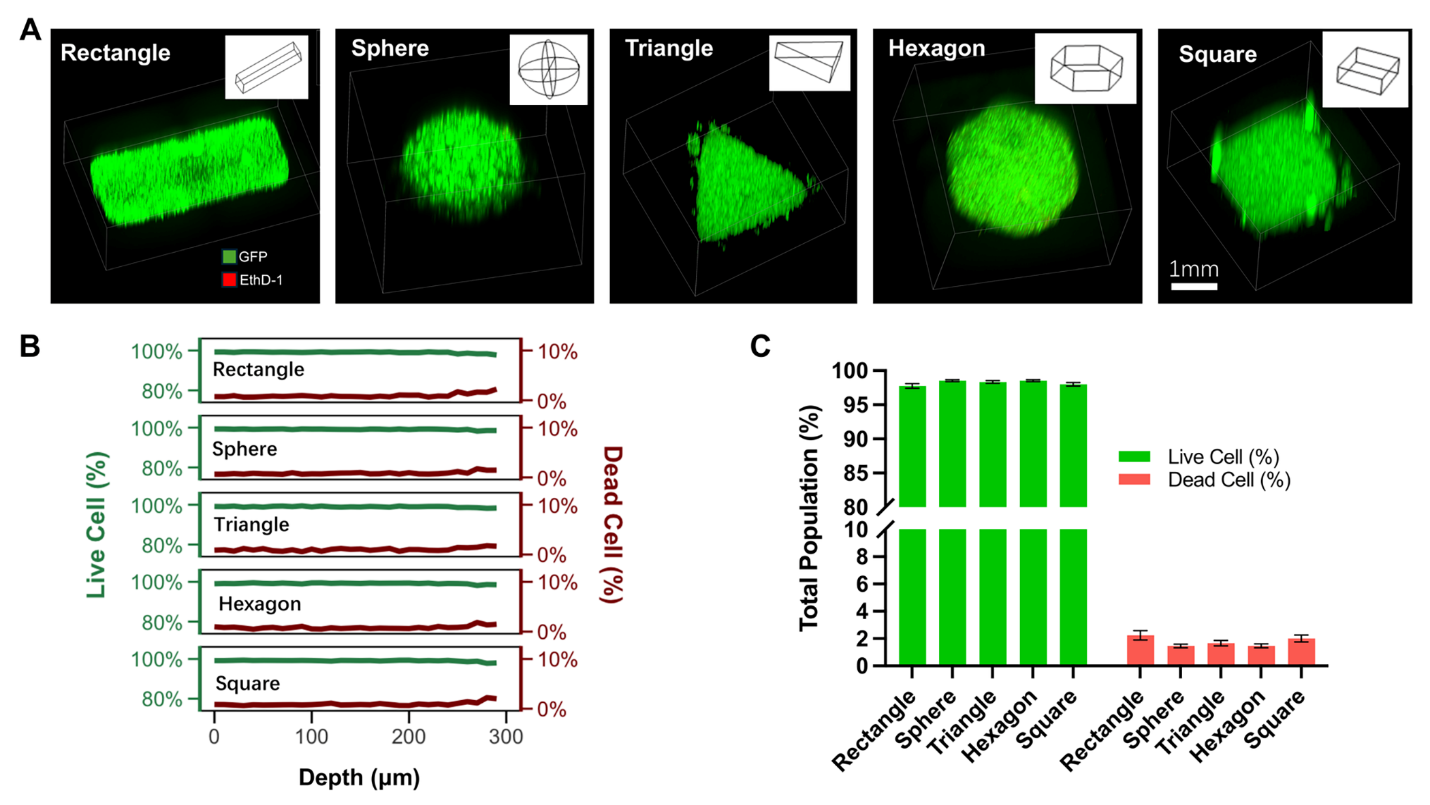
**

**Figure S2. Viability of modular muscle bioconstructs comprising varying geometric shapes. A.** 3D reconstructions of GFP-labeled muscle tissue bioconstructs comprising with different geometries (rectangle, sphere, triangle, hexagon, and square), as imaged by confocal microscopy. Bioconstructs were incubated with ethidium homodimer-1 (EthD-1, red) as an indicator of non-viable cells. Scale bar, 1 mm. **B**. Quantification of live and dead cell percentages across bioconstruct depth of 300µm. **C.** Quantification of cell viability across geometries at 300 µm depth (n=3). Error bars represent mean ± SD.

**
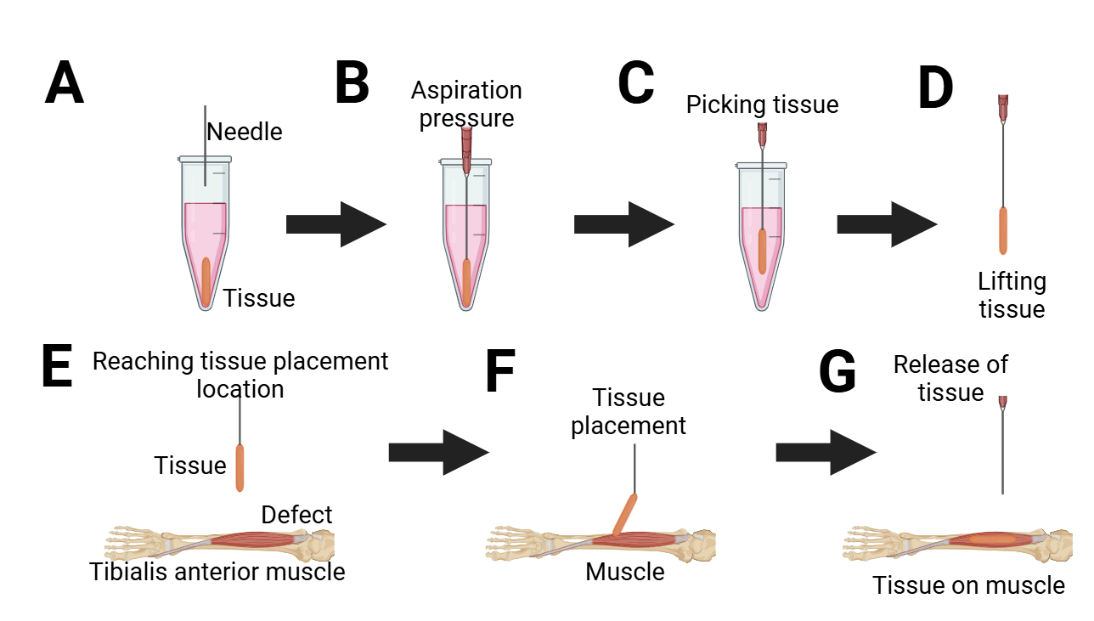
**

**Figure S3. Aspiration-assisted placement of modular muscle tissue into the muscle defect. A-D.** The aspiration needle is placed into the reservoir (A), where aspiration pressure is applied once in contact with the modular tissue (B) and the tissue unit is then aspirated upwards from the reservoir (C-D). **E-G.** The modular tissue is then moved to the muscle defect area of the anesthetized rodent (E), followed by lowering of the needle and release of the aspiration pressure (F), followed by lifting of the bioprinter head away from the muscle defect (H). Created in BioRender. Huang, N. (2025) https://BioRender.com/yiga110

**
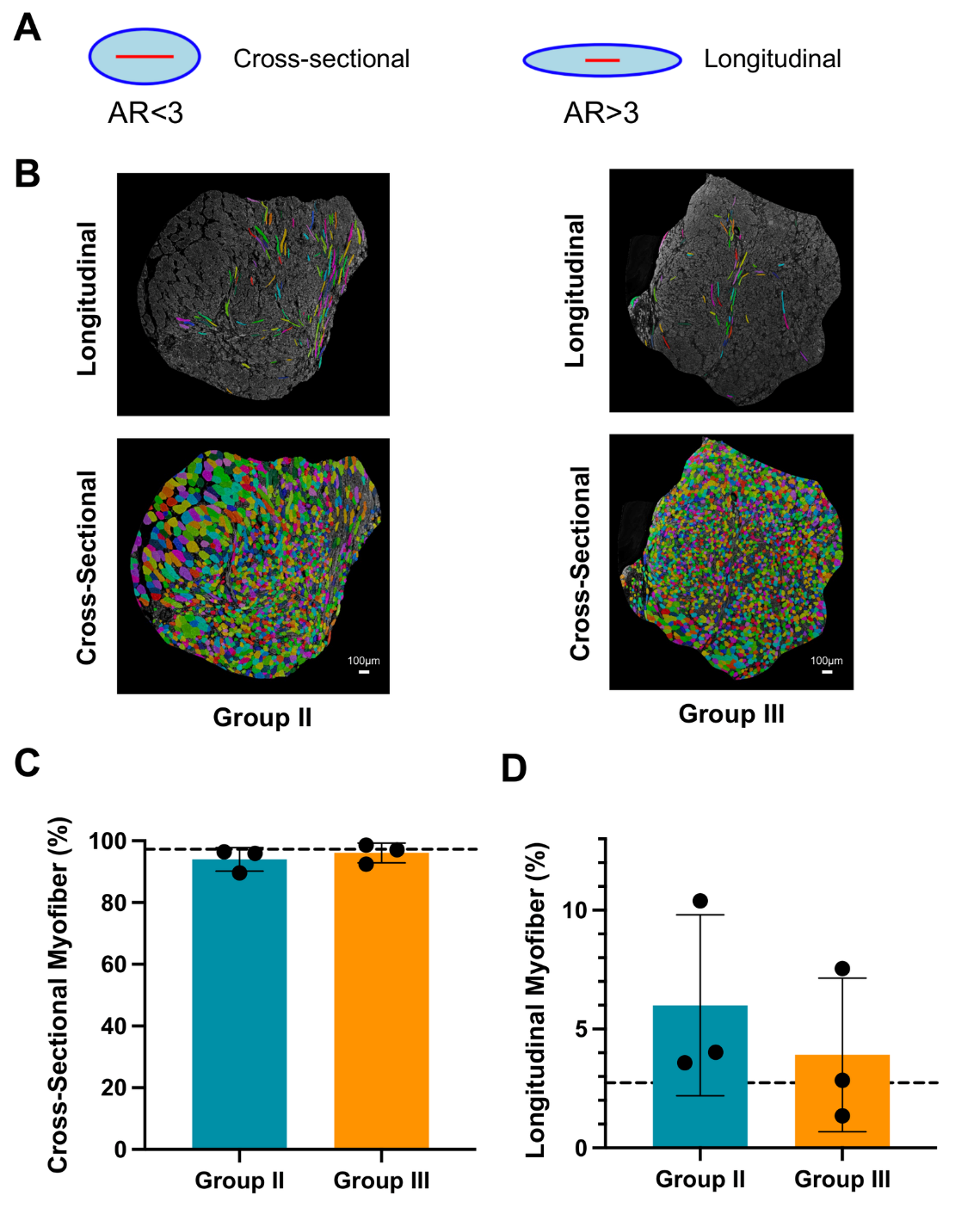
**

**Figure S4. Quantification of myofiber alignment in regenerated muscle following implantation of pre-differentiated tissue bioconstructs. A.** Schematic illustrating classification of myofibers by aspect ratio (AR): fibers with AR < 3 were categorized as cross-sectional, while fibers with AR > 3 were considered longitudinal. **B.** Representative histological images of regenerative regions in volumetric muscle loss (VML) defects treated with scaffold-free tissue constructs pre-differentiated for 2 days (Group II, left) or 4 days (Group III, right) prior to implantation. Orientation analysis was performed on the regenerative portions of explanted tissues, with grayscale images overlaid by longitudinal orientation masks (top) and cross-sectional orientation masks (bottom). Scale bars, 100 µm. **C-D.** Quantification of cross-sectional (C) and longitudinal (D) myofiber fractions in both Group II and Group III. Data represent mean ± SD. Each dot indicates an independent sample; dashed lines indicate baseline levels measured from sham group (n = 3).

**
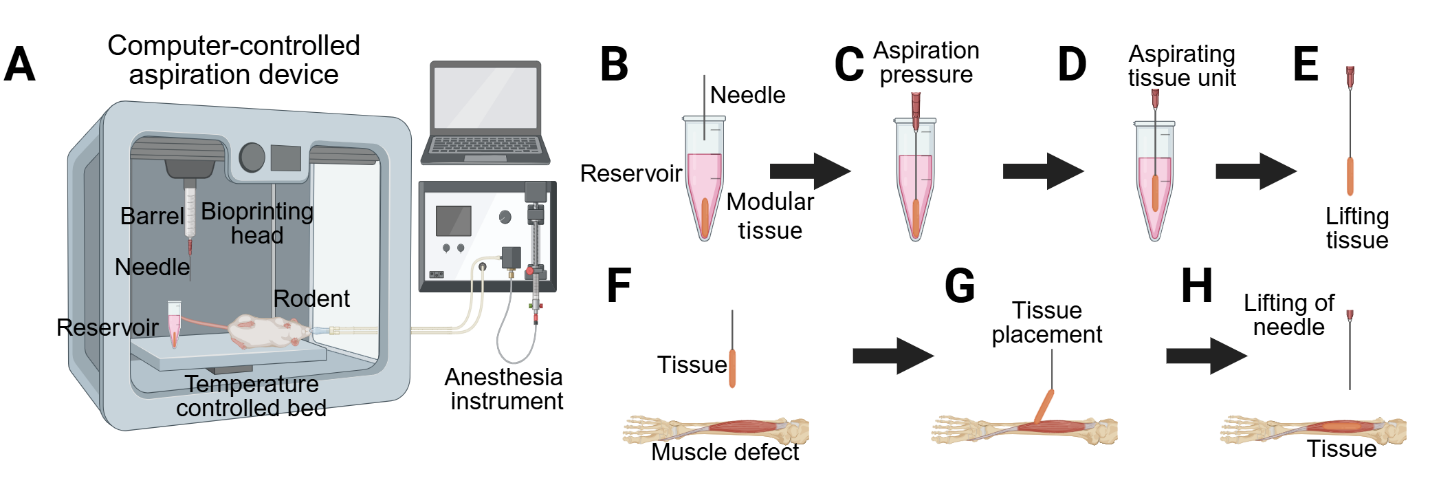
**

**Figure S5. Computer-assisted modular muscle tissue placement into the muscle defect. A.** Aspiration device with computer interface and associated anesthesia instrument. **B-E**. The aspiration needle is placed into the reservoir (B), where aspiration pressure is applied once in contact with the modular tissue (C) and the tissue unit is then aspirated upwards from the reservoir (D-E). **F-H.** The modular tissue is then moved to the muscle defect area of the anesthetized rodent (F), followed by lowering of the needle and release of the aspiration pressure for tissue placement (G), and then lifting of the needle away from the muscle defect (H). Created in BioRender. Huang, N. (2025) https://BioRender.com/n69n441
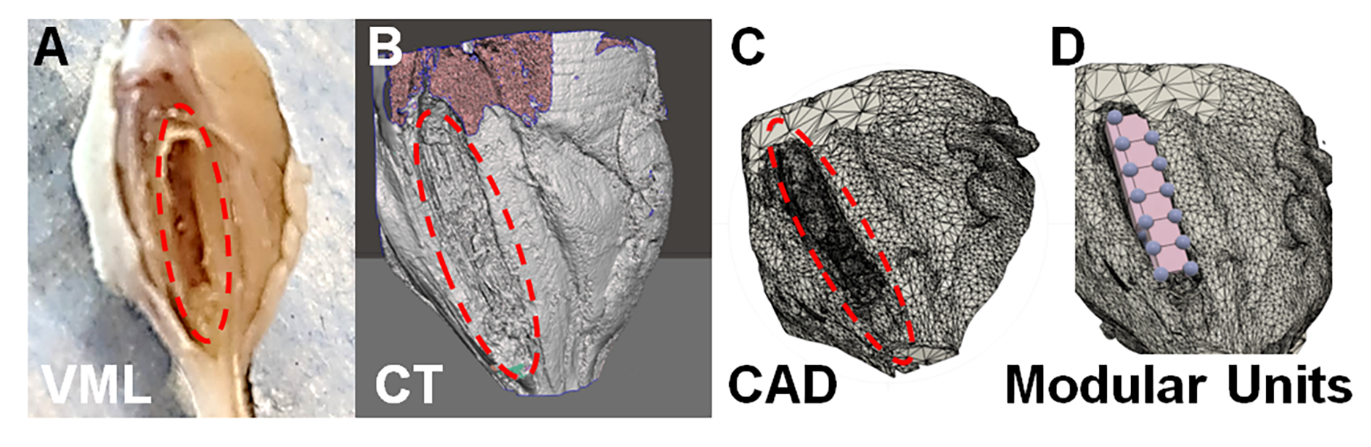
**Figure S6. Futuristic concept of modular tissue unit design and assembly based on patient-specific defect geometries. A-B.** The forward-thinking strategy towards clinical translation of treating customized muscle injury wounds (**A**) consists initially of acquiring high-resolution, 3D computed tomography (CT) scans of the muscle loss defect, as represented by the scan of a mouse muscle defect (**B**). **C.** Then, computer-aided design (CAD) software will create a digital blueprint of the wound region. **D.** Using CAD software and artificial intelligence, it would be feasible to model modular tissue shapes that can be assembled intraoperatively to fit the geometry of the defect. Dotted line denotes muscle defect region.

**SUPPLEMENTAL VIDEO LEGEND**

**Video S1. 3D reconstruction of confocal z-stack depicting the L-shaped C2C12 muscle unit.**

**Video S2. 3D reconstruction of confocal z-stack depicting the L-shaped HMEC vascular unit.**

**Video S3. 3D reconstruction of confocal z-stack depicting the fusion of L-shaped C2C12 (green) and HMEC (red) vascular units after 3 days.**

**Video S4. A toroidal-shaped C2C12 modular tissue constructs was aspirated and then deposited onto the site of the injured muscle by AAIOB.**

**Video S5. A spherical modular muscle construct was bioprinted within a toroidal  tissue construct at the site of muscle injury by AAIOB.**

**Video S6. A cylindrical modular muscle construct was bioprinted adjacent to a toroidal  tissue construct at the site of muscle injury by AAIOB.**
